# Improving the use of expert opinion in disease risk analysis for conservation translocations

**DOI:** 10.1111/cobi.70292

**Published:** 2026-04-24

**Authors:** John G. Ewen, Stefano Canessa, Caitlin E. Andrews, Katie M. Beckmann, Irene Bueno, Deidre K. Fontenot, Caio Kenup, Suzanne Medina, Axel Moehrenschlager, Francesco Origgi, Bruce Rideout, Anthony W. Sainsbury, Thierry M. Work, Claudia Carraro

**Affiliations:** ^1^ Institute of Zoology Zoological Society of London London UK; ^2^ Dipartimento di Scienze e Politiche Ambientali Università degli Studi di Milano Milano Italy; ^3^ Division of Conservation Biology, Institute of Ecology and Evolution University of Bern Bern Switzerland; ^4^ The Nature Conservancy Sacramento California USA; ^5^ Tufts Center for Conservation Medicine Cummings School of Veterinary Medicine at Tufts University North Grafton Massachusetts USA; ^6^ The Royal (Dick) School of Veterinary Studies and the Roslin Institute University of Edinburgh Midlothian UK; ^7^ Bristol Veterinary School University of Bristol Bristol UK; ^8^ Disney's Animals, Science, and Environment Lake Buena Vista Florida USA; ^9^ Division of Aquatic and Wildlife Resources Guam Department of Agriculture Mangilao Guam; ^10^ Conservation Translocation Specialist Group IUCN Species Survival Commission Calgary Alberta Canada; ^11^ Panthera New York New York USA; ^12^ Institute of Animal Pathology (ITPA) University of Bern Bern Switzerland; ^13^ Wildlife Disease Laboratories Institute for Conservation Research San Diego California USA; ^14^ National Wildlife Health Center U.S. Geological Survey Honolulu, Hawaii USA

**Keywords:** assisted colonization, conservation translocation, disease risk assessment, expert judgement, Guam kingfisher, risk analysis, sihek, análisis de riesgo, colonización asistida, criterio de los expertos, evaluación del riesgo patológico, martín pescador de Guam, reubicación por conservación, sihek

## Abstract

Conservation translocations are subject to considerable uncertainty and risk, of which disease is one of the most recognized. To address disease risks, several protocols for qualitative disease risk analysis (qDRA) exist and are used for responsible conservation translocation planning. Existing qDRA protocols usually rely on expert judgment, but they lack quantitative aggregation of elicited estimates, transparent treatment of uncertainty, and explicit comparison of alternative disease risk management pathways. We added these factors to a qDRA for translocation of a charismatic species that is extinct in the wild, the sihek (Guam kingfisher) (*Todiramphus cinnamominus*). We asked a panel of seven independent experts to quantify their judgment of risks of pathogen release, exposure, and consequences for seven pathogen hazards under three translocation management scenarios. Experts suggested that implementing a full biosecurity protocol would reduce risk for several pathogen hazards but would have variable effectiveness (11–65% depending on the hazard). There was substantial uncertainty associated with individual experts and noise among different experts, showing the potential for error if decisions are based on single‐point judgments (i.e., providing judgment of a central tendency without accounting for uncertainty) from one expert or on collaborative consensus of multiple experts, as is current practice.

## Abstract (es)

Mejoras en el uso de las opiniones de expertos para el análisis de riesgo patológico de las reubicaciones por conservación

**Resumen:**

Las reubicaciones por conservación están sujetas a una incertidumbre y un riesgo considerables, siendo las enfermedades uno de los riesgos más reconocidos. Para abordar estos riesgos, existen varios protocolos de análisis cualitativo del riesgo de enfermedad (qDRA) que se utilizan para planificar de forma responsable las reubicaciones por conservación. Los protocolos qDRA existentes suelen basarse en el criterio de los expertos, aunque carecen de una agregación cuantitativa de las estimaciones obtenidas, de un tratamiento transparente de la incertidumbre y de una comparación explícita de las vías alternativas de gestión del riesgo patológico. Incorporamos estos factores a un qDRA para el traslado de una especie carismática extinta en estado silvestre, el sihek (martín pescador de Guam) (*Todiramphus cinnamominus*). Solicitamos a un panel de siete expertos independientes que valoraran los riesgos de liberación de patógenos, exposición y consecuencias para siete peligros patógenos en tres escenarios de reubicación. Los expertos sugirieron que la implementación de un protocolo de bioseguridad completo reduciría el riesgo de varios peligros patógenos, pero tendría una eficacia variable (entre el 11% y el 65%, según el peligro). Observamos una incertidumbre sustancial asociada a los expertos individuales y una variabilidad entre los distintos expertos, lo que resalta el potencial de error si las decisiones se basan en juicios puntuales (es decir, proporcionar una valoración de la tendencia central sin tener en cuenta la incertidumbre) de un solo experto o en el consenso colaborativo de múltiples expertos, como es la práctica habitual.

## INTRODUCTION

Conservation translocations are often used to recover species and ecosystems (Seddon & Armstrong, 2016). Translocations include release of individuals of a species within their indigenous range to reinforce an existing population (reinforcement) or to reestablish a population (reintroduction) (IUCN, 2013). Translocations can also involve moving species outside their indigenous range to support their viability (assisted colonization) or to perform specific ecological functions (ecological replacement) (IUCN, 2013). Planning translocations is typically difficult, because of substantial uncertainty and risk of negative outcomes, where the definition of risk typically reflects both objective probability of negative outcomes and subjective valuation of those outcomes (Canessa et al., 2020; Tulloch et al., 2015). Well‐known risks of translocations include the possibility of inadvertently introducing exotic pathogens and negative effects on translocated animals when they encounter pathogens after release (Beckmann et al., 2022; Cunningham, 1996). These concerns are heightened in an assisted colonization context involving releases outside a species’ indigenous range (IUCN, 2013).

Different methods have been developed to assess disease risks in translocations, classically broken into qualitative, semiquantitative, and fully quantitative approaches (Jakob‐Hoff et al., 2014). In practice, until now disease risks in translocations have been almost exclusively assessed using qualitative disease risk analysis, hereafter qDRA (Davidson & Nettles, 1992, reviewed in Sainsbury & Carraro [2023], although refer to Jakob‐Hoff et al. [2014] for examples of semiquantitative and fully quantitative applications). Proponents of qualitative descriptions state that, where lack of data prevents meaningful quantitative risk assessments, a qualitative approach “readily incorporates lack of precision and it is the best way to use available information to analyse risks and generate the insights needed to make informed decisions about where to focus risk management actions” (OIE & IUCN, 2014). In contrast, there is ample evidence that using qualitative expressions to represent mathematical quantities (e.g., probabilities) can involve substantial linguistic uncertainties and hidden value judgments (Wintle et al., 2019). Overall, quantitative approaches should fully describe uncertainty to avoid giving an impression of false certainty. Qualitative and semiquantitative approaches that rely on expert judgment may be prone to inappropriate scoring and hidden value judgment and should transparently report both within individual expert uncertainty and between‐expert variation (Bojke et al., 2022; Hemming et al., 2018; Morgan, 2014).

All methods of DRA involve a problem description, identification of disease hazards, and risk assessment for identified hazards (OIE & IUCN, 2014). In the risk assessment step, scores are given to the likelihood of release (individual being exposed and infected when translocated), likelihood of exposure (individuals of the same or other species being exposed and infected at the destination), and the expected consequences (biological, environmental, and economic). In qDRA, the scores are expressed on a qualitative scale (typically ranging from verbal expressions of negligible to very high) or with corresponding semiquantitative scores (summarized by OIE & IUCN [2014]; recent examples include Lewis et al. [2019], Vaughan‐Higgins et al. [2021], and Shopland et al. [2022]). An overall risk estimation is then produced, combining these likelihoods and comprising risk posed to translocated individuals, other wild and domestic species, and humans at the destination site (Sainsbury & Carraro, 2023). Sainsbury and Carraro (2023) recommended that in qDRA the relative emphasis on release, exposure, and consequence be summarized in a referenced narrative text. Whenever the overall estimated risk for a given pathogen hazard is not negligible or is above an agreed level of acceptable risk, a disease risk management step is suggested (Jakob‐Hoff et al., 2014; Sainsbury & Carraro, 2023). In qDRA, however, the efficacy of the suggested risk management option is not explicitly presented to a decision maker as a comparison among management options with predictions and their associated uncertainties. The final step in DRA is then implementation and review (OIE & IUCN, 2014).

In the qDRA protocol developed by Sainsbury and Vaughan‐Higgins (2012), the evidence informing hazard identification, disease risk assessments of identified hazards, and disease risk management is typically gathered by literature searches that can take many months to complete. In other variations, the qDRA process is accelerated by combining more rapid literature searches with expert discussion (e.g., Jakob‐Hoff et al., 2014) or by generating simplified scenarios to identify when qDRA is required (e.g., DRAT as described in Jakob‐Hoff et al., 2014; Verant et al., 2022). In all cases, wildlife health experts undertaking the qDRA use this information as a basis to provide their judgments. Although guidelines of the Office International des Épizooties (OIE) and International Union for Conservation of Nature (IUCN) (OIE & IUCN, 2014) indicate that qDRA is appropriate for a first iteration of the process, we are unaware of any instance where this has been followed by quantitative analyses. Rather, the results of qDRA are directly used to inform management. This may reflect a judgment that complexity and uncertainty were insufficient to warrant further analysis beyond qualitative reasoning as described in the OIE/IUCN workbook of procedures for wildlife DRA (Jakob‐Hoff et al., 2014).

Typically, qDRA summarizes evidence and describes risk through a qualitative statement, either from a single wildlife health expert or as a consensus statement from a collaborative group discussion (Jakob‐Hoff et al., 2014; Sainsbury & Carraro, 2023). Qualitative descriptions of risk involve personal interpretations and implicit judgments that are difficult to identify (Wintle et al., 2019). Group discussions that seek consensus are prone to linguistic uncertainty, inconsistencies, and groupthink biases (Morgan, 2014; Wintle et al., 2022). To address these common biases affecting expert judgment, formal elicitation methods have been developed that seek independent judgments from multiple experts following appropriate training, and include structured feedback and updating (e.g., Hemming et al., 2018). Experts express their judgments with a transparent description of their own uncertainty (Hemming et al., 2018), possible even when using qualitative constructed risk scales (Zapata‐Vázquez et al., 2014). Carefully structured expert elicitation helps reduce commonly encountered biases within single experts and across group discussions (Bojke et al., 2022; Hemming et al., 2018; Morgan, 2014), allows aggregation and comparison of viewpoints, and provides a transparent way to assess individual expert uncertainty as well as variation (noise) among experts (caused, for example, by disagreements about the system's structure and function). Current qDRA methods could more effectively incorporate such practices in the treatment of expert opinion.

We used a proposed assisted colonization of the sihek (*Todiramphus cinnamominus*) (Guam kingfisher), which is extinct in the wild, as a case study to reveal expert uncertainty and noise among experts in qDRA. In doing so, we sought to provide modifications to qDRA for wildlife conservation translocations that make it semiquantitative, that elicits judgments across multiple experts, and that more directly compares the effectiveness of risk management alternatives. We focused on pathogen hazards and on the risk assessment and risk management steps of DRA. There is limited knowledge about sihek disease, and translocation‐associated disease risks are of particular concern given sihek are managed in zoos that hold multiple avian species (and their pathogens) from multiple places (Vernet et al., 2024). We asked experts how the risk of introducing pathogens to new habitat would change across three alternative translocation pathways with differing levels of disease risk management.

## METHODS

### Source and destination sites and populations

Sihek are endemic to Guam but were extirpated from the wild around 1988. From 1984 to 1986, an ex situ population was founded from 16 individuals, which has since grown to 137 adults as of June 2024 (Trask et al., 2021). The ex situ population is housed across 25 facilities, including 24 Association for Zoos and Aquariums (AZA) institutions across North America and one facility at the Department of Agriculture on Guam. Sihek are housed in dedicated enclosures separated from other species and held individually when not breeding. However, all AZA institutions with sihek are zoos that hold many species from multiple origins, in addition to hosting local wild‐living species that inhabit the zoological grounds.

The proposed translocation destination is Palmyra Atoll, a 235‐ha isolated set of 28 islets in the Northern Line Islands, about 1600 km south of Hawaii (5°53ʹ60ʺ N, 162°60ʹ11ʺ W), with no historical evidence of kingfishers but with 20 species of seabirds and shorebirds (Appendix S1). Terrestrial vertebrates comprise two gecko species (*Lepidodactylus* sp. nov. and *Lepidodactylus lugubris*). Competent arthropod vectors for some pathogen hazards are present on Palmyra Atoll, including *Culex* mosquito species (Appendix S1). Little else is known about pathogens on Palmyra Atoll.

### Pathogen hazards

Seven pathogen hazards were assessed for disease risk in the sihek translocation. For each of the seven pathogen hazards, a disease risk assessment was initiated by C.C., a specialist, who reviewed and summarized in referenced narrative text available evidence justifying the hazard across release, exposure, and consequence (Appendix S2) (methods detailed in Sainsbury & Vaughan‐Higgins [2012]). Up to this point, we followed the normal qDRA protocol. However, C.C. did not provide estimates of likelihood of release, exposure, or consequence, as is normally done with qDRA. Instead, the unfinished risk assessment was used as a starting point for further, more structured semiquantitative risk assessment (see below).

### Alternative conservation translocation pathways for sihek

**FIGURE 1:**
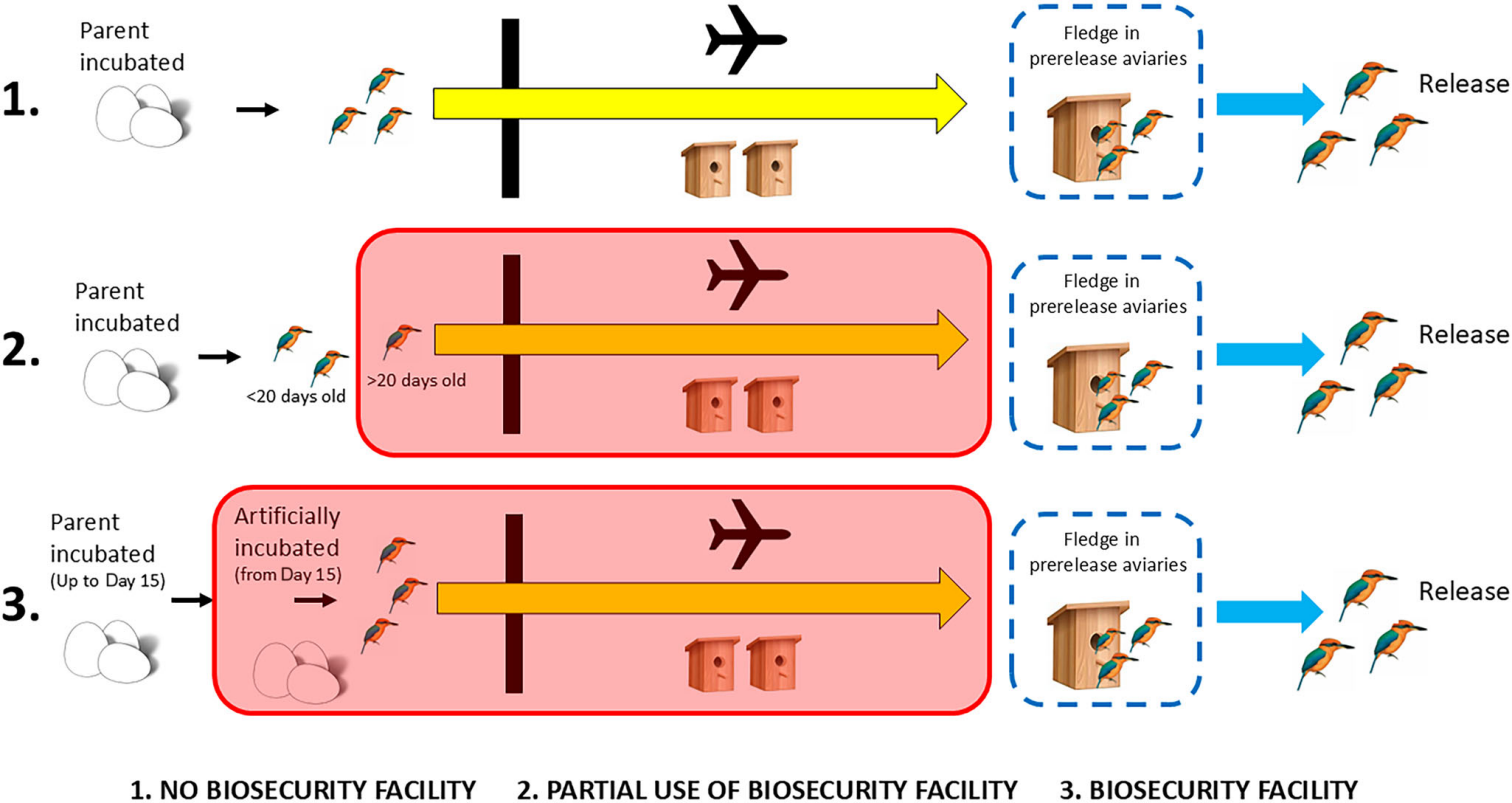
Alternative translocation pathways under consideration by risk assessors in a conservation introduction of sihek (*Todiramphus cinnamominus*) (red rectangles, when sihek are in a purpose‐built biosecure facility; black bars represent geographic [oceanic] barrier between source and destination sites).

Based on the seven pathogen hazards, we developed three alternative translocation pathways that differed in the extent of biosecurity prior to placement in prerelease aviaries. These three broadly different alternatives reflected the desire for a robust, purpose‐built biosecurity pathway isolated from other zoo operations given the context of this conservation translocation (Vernet et al., 2024) and the preference expressed by some managers for eggs to remain at host institutions for parental incubation and hand rearing, following standard protocols for this species (no red shading in Figure 1). All alternative pathways included hand rearing because this method increases chick survival rates (Trask et al., 2021) and the group agreed it would reduce the risk of parent–chick disease transmission.

All translocation pathways will include flying 20 sihek to Palmyra Atoll via Honolulu airport as nestlings (27–30 days old, fully developed, feathered, and thermoregulating) or young fledglings (32–35 days old). At Palmyra Atoll, sihek will be held in prerelease aviaries for about a month before release. Any biosecurity measures cease when birds enter prerelease aviaries. The following three biosecurity alternatives build on this general pathway base.

Alternative 1 is no use of a biosecurity facility. Parent‐incubated eggs will be collected for hatching and hand rearing just prior to pipping (based on an incubation period of 22–23 days). Nestlings will be hatched and hand reared at the host institution (Figure 1). At 20 days old, each nestling will receive a clinical examination by the host institution's veterinarian (Appendix S3), including hazard screening tests (Appendix S3 contains details on each infectious hazard). Only nestlings assessed as healthy and testing negative for these infectious hazards will be shipped to Palmyra Atoll. Transfer crates will not include barriers to blood‐sucking vectors (mosquitoes, mites, midges, flies).

Alternative 2 is partial use of biosecurity facility. Parent‐incubated eggs will be collected for hatching and hand rearing just prior to pipping. Nestlings will be hand reared at the host institution. Once nestlings are old enough to be transported (about 20 days old), they will receive a clinical examination (Appendix S3). If they are deemed healthy, they will be shipped to the biosecure facility (see Alternative 3 for full description) (Figure 1). After approximately 2 weeks in this facility, they will undergo hazard screening tests (Appendix S3). If these tests are negative, they will be shipped to Palmyra Atoll in blood‐sucking‐vector (mosquitoes, mites, midges, flies) proof crates.

Alternative 3 is use of a biosecurity facility. Fertile eggs will be harvested from breeding pairs after two thirds of the incubation period (at about Day 15 of incubation) and shipped to a biosecure facility where they will be incubated, hatched, and hand raised (Figure 1). The facility will be blood‐sucking‐vector (mosquitoes, mites, midges, flies) proof, and incubators and hatcheries will be dedicated only for use with sihek. Stringent biosecurity barriers will be in place at the entrance to the facility, including a disinfectant footbath, and only dedicated personnel will be permitted within the facility wearing dedicated clean clothing (overalls and/or lab coats). At 20 days old, each sihek nestling will receive a clinical examination combined with hazard screening tests (Appendix S3). Only birds deemed healthy and test negative will be shipped to Palmyra Atoll in blood‐sucking‐vector (mosquitoes, mites, midges, flies) proof crates.

### Disease risk estimation

We elicited from seven experts their judgments on disease risks arising from each pathogen hazard under each alternative translocation pathway. Experts were selected because they either worked with sihek (*n* = 3), worked on other avian qDRAs for conservation translocations (*n* = 4), or were experienced wildlife veterinarians (all 7). We purposefully sought diversity through gender balance (four female, three male) and geographic spread (United Kingdom, 3; United States, 3; European Union, 1) and allowed online group discussion at reasonable times. Seven experts is a minimum adequate number of experts, as recommended by Gregory et al. (2012). All experts were provided with the hazard sheets prepared by C.C. (see above) for review prior to elicitation. We followed the IDEA (investigate, discuss, estimate, aggregate) protocol (Hemming et al., 2018), running the elicitation online with an integrated ShinyApp web application and online discussions, although we were unable to run a formal training session for experts prior to the elicitation. This deviates from recommended best practice and may therefore influence within‐expert uncertainty and between‐expert noise in first‐round judgments.

Each judgment was elicited using a 4‐point method in which we asked for the low and high bounds, a best guess, and finally an estimate of the expert's confidence that the true value would fit within the range provided. The ShinyApp web application included a description of the alternative translocation pathways, the risk assessment information sheet for each pathogen hazard, and a graphical and tabulated summary of prevalence, virulence, and sample size data from our previous literature review (Appendix S4). After reviewing this information, each expert was asked to provide quantitative opinions on four parameters: the probability that a sihek will have become exposed and infected with the pathogen hazard by the time it is released on Palmyra Atoll (Parameter 1); given at least one infected sihek has been released, the number of sihek that will die in the first year following their release because of disease induced by that pathogen hazard (additional to background mortality) (Parameter 2); given at least one infected sihek has been released, the probability of the pathogen hazard causing a disease epizootic in other species at the destination (Parameter 3); and given the release of at least one infected sihek, the probability of the pathogen hazard becoming enzootic at the destination (Parameter 4). The question associated with Parameter 4 reflected a preference to avoid accidental establishment of novel pathogens in conservation introductions.

Given the known global distribution of the pathogens *Aspergillus fumigatus*, *Chlamydophila psittaci*, and *Mycobacterium* spp., we assumed they could already be present on Palmyra Atoll. Therefore, for these hazards, Parameter 2 was modified to include encountering the pathogen hazard at the destination, and Parameters 3 and 4 were removed for simplification under the assumption that the addition of such a small number of founders would not alter a disease epizootic on the island. Similarly, the host‐specific pathogen hazard *Isospora* spp. was not assessed for Parameters 3 and 4. To provide a reference baseline for additional mortality in Parameter 2, we stated that 47% (nine of the 20) of released sihek would be lost in the first year as a result of other natural causes. This was based on a midpoint between survival rate estimates of the same age class in captive sihek (Trask et al., 2021) and in a wild declining population of Pohnpei kingfisher (*Todiramphus reichenbachii*) (Kesler & Haig, 2007). We asked experts to treat each pathogen hazard independently and not assume coinfection.

During the elicitation, the web application allowed experts to visualize their estimates in real time as probability distributions and waffle plots, which translated probabilities into visual depictions of the expected number of infected and dead sihek. Integration of real‐time feedback of quantitative estimates was provided to help experts obtain a clear understanding of how 4‐point elicitation captures their opinions and what this means in real terms for fates of released sihek and the recipient ecosystem. Experts were then allowed to visualize each other's initial estimates (in anonymous form), discuss with each other during facilitated online meetings, and revise their estimates after these discussions if they so wished. We rescaled all estimates from the final round of elicitation to 100% confidence and fitted a beta‐PERT (program evaluation and review technique) distribution for each expert's opinion following recommendation from Hemming et al. (2018). We then aggregated opinions across experts through linear pooling, where the probability density of the aggregated distribution is the average of the probability densities across experts.

We combined the aggregated opinions for the probability of infected sihek arriving on Palmyra Atoll and for the consequence for each biosecurity alternative with a decision tree. We reported the mean expected values for each hazard on sihek additional mortality, probability of causing an epizootic, and probability of the pathogen becoming enzootic on Palmyra Atoll. We resampled the tree 10,000 times, resampling each parameter's probability distribution, to obtain an empirical distribution of all expected outcomes, which we summarized using mean and 95% confidence intervals.

### Noise audit of experts

Noise is the variation among experts in the expressed estimates. Using formal 4‐point elicitation, a first measure of noise is provided by the comparison of the fitted probability distributions for individual experts’ estimates. To further assess noise, we calculated the noise index as detailed in Kahneman et al. (2021):

$$I=\sum_{1\leq i\leq j\leq n}^{N}\frac{\left|\hat{e_{i}}-\hat{e_{j}}\right|}{\left(\hat{e_{i}}+\hat{e_{j}}\right)/2}\times \frac{1}{N},$$

where *e_i_* and *e_j_* are elicited opinions of a pair of experts *i* and *j*, and *I* is a measure of mean disagreement among pairs of experts (how different each pair of opinions is compared with their average). We calculated the mean difference of pairwise expert values per elicited judgment and report mean disagreement by pathogen hazard; by the probability of a sihek arriving infected; by additional mortality; and, where relevant, by the probability of the pathogen hazard causing an epizootic and the probability of it becoming enzootic on Palmyra Atoll.

## RESULTS

### Risk estimation

**FIGURE 2:**
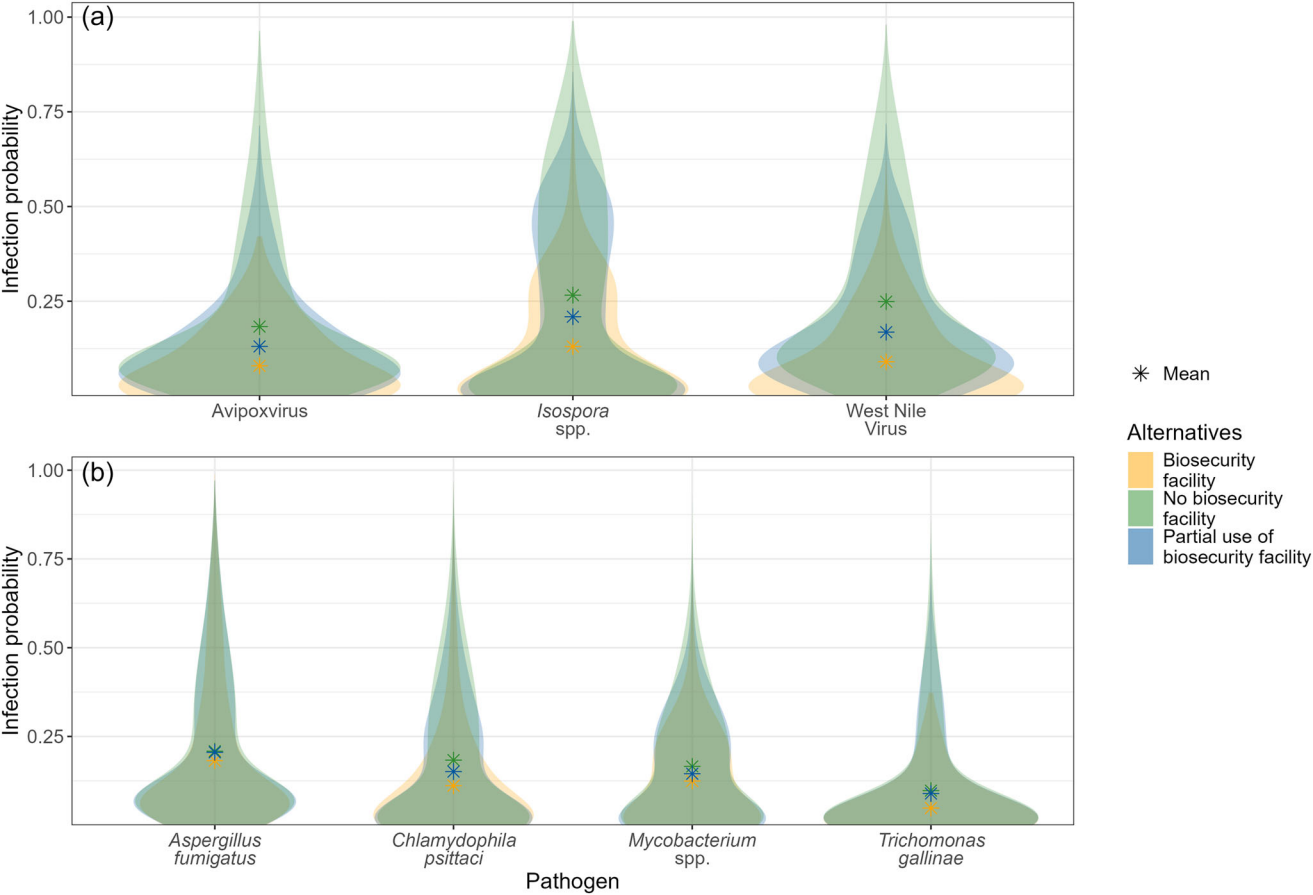
Aggregated expert opinions for probability that at least one sihek (*Todiramphus cinnamominus*) being translocated to Palmyra Atoll arrives infected with a pathogen under three biosecurity pathways (yellow, biosecurity measures; blue, partial biosecurity measures; green, no biosecurity; asterisk, mean opinions across experts; violins, uncertainty in these opinions): (a) experts judge that increased levels of biosecurity will reduce risk of infection and (b) experts judge that there is little change in risk from a pathogen with increasing levels of biosecurity.

Aggregated judgments from across the seven experts showed three broad patterns of perceived disease transmission risk and potential for mitigation (Figure 2). For the pathogen hazards West Nile virus and avipoxvirus, there was a high perceived probability that a sihek would have become exposed and infected before release and belief that the increasing levels of biosecurity would be highly effective at reducing this infection probability (Figure 2a). For the carrier hazard *A. fumigatus*, there was a high perceived probability that a sihek would become exposed and infected but weaker belief that this would be reduced by increasing levels of biosecurity (Figure 2b). For the remaining pathogen hazards, there was lower perceived probability of infection and the experts considered that using a biosecure facility would reduce this probability by about half relative to not doing so (Figure 2).

**FIGURE 3:**
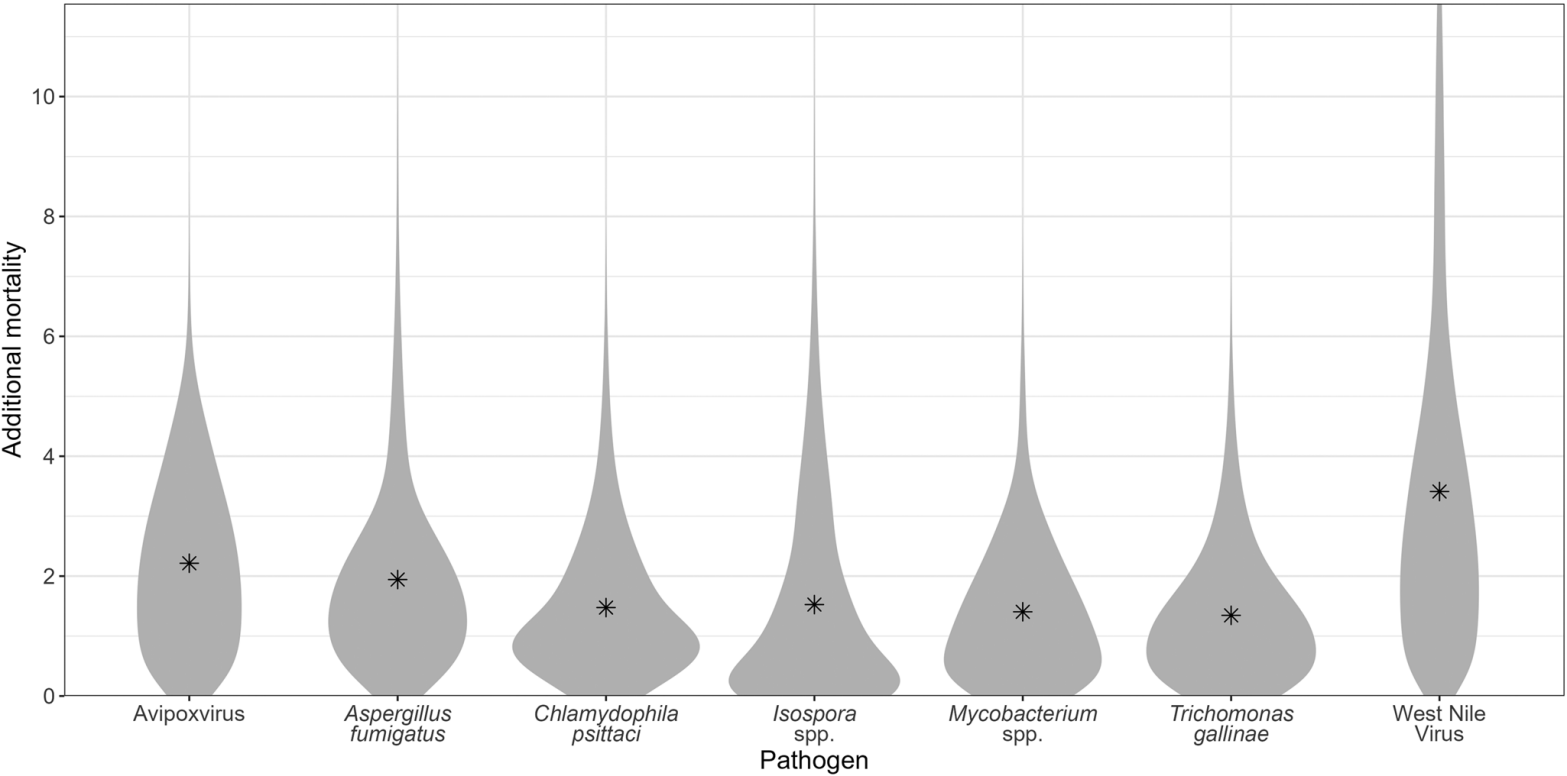
Aggregated expert opinions on additional mortality of released sihek (*Todiramphus cinnamominus*) (out of the 11 birds predicted to remain from a total release cohort of 20 birds, given background mortality) that could occur if a pathogen is cointroduced or encountered at the destination (stars, means; violins, uncertainty in expert opinions).

**FIGURE 4:**
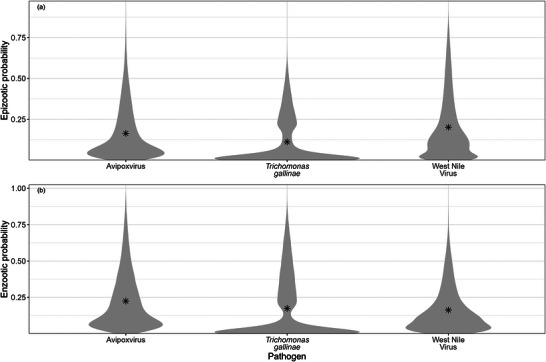
Aggregated expert opinions on the probability that each pathogen that is absent from Palmyra Atoll if cointroduced with sihek (*Todiramphus cinnamominus*) would cause an epizootic in other (a) wildlife or become (b) an enzootic pathogen in wildlife on Palmyra Atoll (stars, mean opinion across experts; violins, uncertainty).

The experts believed pathogen hazards could result in added mortality among released sihek. Experts believed this additional mortality in the first 12 months after release ranged from a low of 1.5 (aggregated mean; aggregated 95% confidence limit [CL] 0.0–6.2) additional released sihek dying from *Isospora* spp. to a high of 3.4 (0.20–10.7) additional released sihek dying from West Nile virus (Figure 3). Second, pathogen hazards that were absent from Palmyra Atoll and carried by sihek may spill over to other species at the destination and cause an epizootic. Experts believed the probability of this occurring ranged from an aggregate mean of 0.011 (95% CL 0.000–0.479) for *Trichomonas gallinae* to about 0.202 (95% CL 0.005–0.690) for West Nile virus (Figure 4a). Third, pathogen hazards absent from Palmyra Atoll may spill over and become enzootic in the destination environment. Experts believed the probability of this occurring ranged from a low of 0.174 (0.000–0.695) for *Tr. gallinae* to a high of 0.225 (0.018–0.706) for avipoxvirus (Figure 4b). In all cases, there was substantial uncertainty expressed across experts.

**FIGURE 5:**
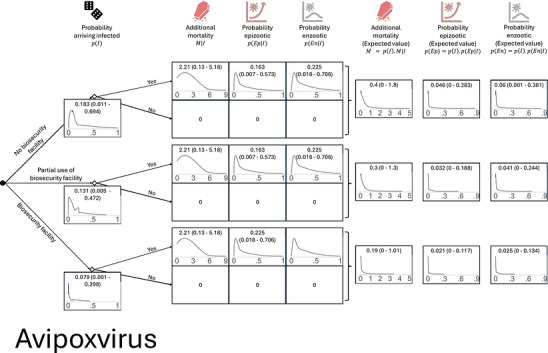
Decision tree showing the utility of three different levels of biosecurity on reducing the probability of sihek (*Todiramphus cinnamominus*) arriving to Palmyra Atoll infected with the *Avipoxvirus* pathogen and the ramifications of this pathogen arriving on the number of additional deaths of released sihek (additional mortality column), on the probability of causing an epizootic at the destination (probability epizootic column), and on the probability of establishing a new pathogen as enzootic at the destination (probability enzootic column). Management ramifications on objectives are mean expected values (EVs) derived from resampling (10,000x) from the uncertain probability of arriving infected multiplied by the resampled uncertain outcome if that happened. Distributions of elicited quantities are aggregated across experts through linear pooling, and values are presented as mean and lower and upper confidence intervals (calculated as the 2.5% and 97.5% quantiles of the aggregated distribution). Similar decision trees for the other six hazards are in Appendix S5.

We combined the expert judgment of the probability and consequences of infected sihek arriving on Palmyra Atoll with a decision tree (Figure 5). Full biosecurity was judged to reduce negative outcomes for all hazards, although the magnitude of this reduction varied across pathogen hazards. For example, compared with no biosecurity, full biosecurity reduced the predicted risk from disease by 65% for West Nile virus but only by 11% for *A. fumigatus* (Figure 5). The decision tree also showed large overlap in predicted consequences among the three alternatives, indicating the substantial uncertainty around predictions.

### Noise audit

Our noise audit showed there were substantial differences among experts in their judgments. Distributions were largely unimodal, except for minor secondary modes for the probability of infection by *Isospora* spp. (Figure 2) and for mortality and probability of epizootic by *Tr. gallinae* (Figures 3 & 4). However, there was still considerable dispersion. Comparisons among experts for each pathogen hazard showed that best guess judgments differed between 94% for *A. fumigatus* and 151% for *Tr. gallinae* (Table 1). The level of agreement did not change whether experts were judging a probability or the number of additional sihek dying in the first‐year post release, with mean noise values for best guess judgments across the four parameters ranging from 106% to 132% (Table 1). Between the two rounds of elicitation, noise increased for nine parameters, decreased for seven, and did not change for four out of 20 parameters. The average change across parameters was 4.79 percentage points (Table 1).

**TABLE 1:** Mean difference from pairwise comparisons of seven expert opinions on disease risk following two rounds of elicitation with the IDEA (investigate, discuss, estimate, aggregate) protocol and change in noise between rounds.

Quantity	Infectious hazard	Difference (%)		Change in noise
		Round 1	Round 2	
Additional mortality	Avipoxvirus	64.8	94.7	29.8
	*Aspergillus fumigatus*	64.8	93.3	28.6
	*Chlamydophila psittaci*	98.7	84.4	−14.2
	*Isospora* spp.	150.0	144.4	−5.6
	*Mycobacterium* spp.	155.5	144.4	−11.1
	*Trichomonas gallinae*	126.6	120.0	−6.7
	West Nile virus	69.0	120.0	51.0
Infection probability	Avipoxvirus	120.1	126.2	6.1
	*Aspergillus fumigatus*	91.9	99.9	8.0
	*Chlamydophila psittaci*	149.0	149.0	0.0
	*Isospora* spp.	168.7	168.7	0.0
	*Mycobacterium* spp.	130.9	151.2	20.4
	*Trichomonas gallinae*	164.2	160.8	−3.4
	West Nile virus	120.5	120.5	0.0
Epizootic probability	Avipoxvirus	121.3	106.8	−14.5
	*Trichomonas gallinae*	162.2	162.2	0.0
	West Nile virus	108.6	115.6	7.0
Enzootic probability	Avipoxvirus	90.0	91.9	1.9
	*Trichomonas gallinae*	157.6	154.3	−3.3
	West Nile virus	110.8	112.6	1.8

*Note*: All values pertain to best guesses by the seven experts.

## DISCUSSION

Combining the current approaches to qDRA with best practice elicitation methods for assisted colonization of sihek, seven pathogen hazards were evaluated in terms of expert belief in the probability of being present in translocated sihek and of the consequences of this event for the sihek themselves and for the recipient ecosystem across risk management alternatives. Although using a biosecurity facility was judged to reduce these risks, these predicted improvements were often relatively minor, and there were substantial uncertainty and noise within and across judgments, highlighting that summaries (qualitative or quantitative) from single experts or group consensus are unlikely to accurately represent the true risk. We sought to provide a more nuanced summary of perceived disease risks for decision makers by modifying a commonly used qDRA approach in two ways. First, we consulted multiple experts and aggregated their judgments following a formal elicitation process. Second, we asked experts to explicitly provide their judgments on the effectiveness of multiple alternative options for risk management.

Disease risk analysis protocols draw strongly on available evidence but most involve a degree of expert judgment and are therefore vulnerable to heuristics and cognitive biases that formal elicitation methods seek to reduce (Burgman et al., 2011; Martin et al., 2012). In particular, many current qDRAs do not clearly represent uncertainty in a single expert's assessment (because risk is assigned to a single qualitative category) or noise among experts where multiple experts are consulted (because discussions are summarized into a single overall qualitative category). Drawing on the opinions of multiple experts helps understand noise and uncertainty and reduce bias (Martin et al., 2012), particularly when following a structured approach (Burgman et al., 2011).

Groups are obviously not immune from the biases of individuals and specific groupthink dynamics (Turner & Pratkanis, 1998). We adopted multiple best practices to reduce these biases. First, rather than a single judgment of risk on a qualitative constructed scale, we decomposed our risk assessment into multiple quantitative estimates of probabilities and outcomes (Arkes et al., 2010). The IDEA protocol, based on a modified Delphi approach, is used to counter group biases by emphasizing anonymous individual judgment, allowing feedback and revision (Hemming et al., 2018). To standardize access to information and reduce noise, we provided all experts with baseline knowledge before the elicitation. Estimating probabilities and numbers on natural scales, instead of constructed scales, helped us reduce linguistic uncertainty (Wintle et al., 2019). Visual guides (such as translating mortalities into actual number of birds affected) and facilitated discussion helped us minimize the cognitive challenges of estimating probabilities (O'Hagan et al., 2006). Four‐point elicitation that includes an estimate of uncertainty also helps reduce overconfidence (Speirs‐Bridge et al., 2010). Training and calibration of experts before the elicitation can further reduce process uncertainty and improve performance. In our case, the lack of such best practice steps might have hindered expert performance and contributed to the persistence of noise at least in the initial round. Although the lack of substantial differences in noise and uncertainty before and after the discussions suggests this was not a primary driver of noise, we will seek to develop a reliable training and calibration approach for future elicitations.

We elicited complex probabilities of higher order outcomes, such as epizootic outbreaks as a result of bird infection and subsequent pathogen introduction, which required complex mental models and likely influenced uncertainty and noise, as experts noted during discussions. This is less a result of our specific approach than it is a necessity of most translocation DRAs, which focus precisely on such higher order outcomes. This challenge simply reinforces the need to represent difficult expert judgments more transparently, breaking the most complex ones into intermediate steps to reduce uncertainty and noise (Arkes et al., 2010; Kahneman Sibony & Sunstein et al., 2021). Quantitative elicitation lends itself to such disaggregation better than qualitative approaches. For example, a more complex elicitation could involve parameterization of an epidemiological model with full propagation of uncertainty, which would then be used to estimate higher order outcomes (Canessa et al., 2018).

Elicitation from multiple experts, although beneficial in terms of accuracy, requires additional considerations about aggregation. In qDRA protocols that rely on verbal categories, it would not be intuitive how to aggregate, for example, judgments of “negligible” and “high” risk without adding further subjectivity. Although a simple solution is often to convert such expressions to numerical scores, the OIE and IUCN guidelines caution against this practice, which is often highly arbitrary, especially when followed by mathematical manipulation that can hide important value judgments (Jakob‐Hoff et al., 2014). Aggregation of verbal or semiquantitative constructed scales is still possible but might require different approaches such as Bayesian updating (Hartley & French, 2021) or fuzzy logic (Siler & Buckley, 2005). Conversely, by directly expressing quantitative estimates of risk as natural values (numbers of individuals and probabilities), we were able to directly compare expert opinions, aggregate them, and evaluate uncertainty and noise more transparently than would have been possible with verbal statements and semiquantitative scores.

We found substantial noise within and across experts in the aggregated distribution of individual judgments and in the additional calculation of pairwise comparisons. Our audit of among‐expert variation revealed high levels of noise in their best estimates, ranging from a low of 95% to as high as 151% mean differences among experts for a given opinion. In other words, the pairwise difference between experts was at least as big as their average. Kahneman et al. (2021) report that for a case study of financial judgments, executives considered noise levels in the 40–60% range as not tolerable. Such noise may reflect a mix of linguistic uncertainty in the information being elicited and of different mental models informing those estimates. In our study, similar levels of noise persisted even following discussion and updating; in some cases, noise even increased in the second round of discussion, after additional information and new mental models were shared. This suggests that noise likely reflected persistent different mental models and opinions, rather than linguistic uncertainty that was clarified during the discussion. Exposing such differences and reducing linguistic uncertainty are key purposes of the IDEA protocol. Formal training of experts before the elicitation might have further accelerated this improvement. Transparent reporting of noise within and across experts provides challenges to decision makers but does allow them to constructively articulate their risk tolerance to uncertainty and become more accountable for the decisions they make (Stirling, 2010). Despite substantial uncertainty in the sihek case study, the decision was made to continue with the assisted colonization. Representing uncertainty transparently also opens the possibility to design learning that could reduce it to better satisfy the risk tolerance of decision makers.

We have no reason to believe that the uncertainty and noise we found are specific to sihek. On one hand, all sihek are held under human care in zoos, and their health is closely monitored (Trask et al., 2023), possibly providing more information than analogous DRAs for conservation translocations of free‐living wild populations. On the other hand, the lack of a free‐living wild population means there is no baseline knowledge of parasite occurrence and prevalence in free‐living wild sihek, forcing experts to rely on extrapolation from other species and areas. Most translocations face such conditions; therefore, high levels of uncertainty and noise may be common in qDRA but remain hidden because of the current approach's limitations.

Noise does not always indicate error, and absence of noise is not absence of error. Some degree of noise is inevitable and even desirable when one seeks to capture a diversity of judgments. In this sense, noise is a form of uncertainty that may arise from a poorly defined problem, a stochastic future, or insufficient knowledge (Regan et al., 2002). Practitioners could address it by collecting additional information, by adopting decision‐analytic methods to act in the face of uncertainty, or by implementing actions and learning in an adaptive management framework (Gregory et al., 2012). However, the necessary first step to such practices is to report and reflect on this noise. Failure to do so has been detrimental to outcomes across various fields ranging from human medicine to criminal justice (reviewed in Kahneman et al. [2021]). Conservation should follow the experience of other disciplines, such as human medicine, that continue to advance through a growing variety of approaches, including better expert training (e.g., Phillips et al., 2004), use of multiple experts (e.g., Barnett et al., 2019), and reporting noise in standardized ways so that a realistic summary of uncertainty is provided to decision makers (e.g., use of the kappa statistic) (Sim & Wright, 2005; Viera & Garrett 2005). We simply reported noise, knowing that our general best practices can already reduce it to some extent (Armstrong, 2001). Different aggregation methods might be used to reduce noise more actively, for example, by weighting down the less informative experts (Cooke, 1991). We decided against this because facilitators were unsure of the criteria to use for calibration and weighting, given the lack of data on postrelease sihek on Palmyra, and were concerned of adding their own biased assumptions against an unclear benefit of weighting (Bolger & Rowe, 2015).

Finally, our elicitation explicitly assessed various options for disease risk management. Such explicit comparisons provide ideal guidance for decision‐making but remain uncommon in most translocation studies, including but not limited to DRA (Taylor et al., 2017). Eliciting the judgment of experts not only on disease risks but also on mitigation options helped us close this gap. Tools like decision trees allow experts and managers to visualize the sequence of choices and stochastic events that would lead to a given higher‐order outcome (e.g., preventing establishment of a new pathogen in Palmyra Atoll) and to calculate the uncertainty surrounding those outcomes. For assisted colonization of sihek to Palmyra Atoll, experts indicated that the risks from West Nile virus and avipoxvirus might be reduced through increasing levels of biosecurity, whereas this was not the case for *A. fumigatus*. Direct estimates of mitigation effectiveness with their associated uncertainty can also help understand different risk tolerances (Canessa et al., 2020). This integration of assessing risk changes in response to management options has provided powerful insights for DRAs in other contexts (e.g., Cook et al., 2021) but is novel for DRAs supporting conservation translocations. We do not distinguish source institutions in this DRA, although this could be done in future iterations with consideration of uncertainty in pathogen presence given available health records.

Disease is one of the key risk categories highlighted in the IUCN Guidelines for Reintroductions and Other Conservation Translocations (IUCN, 2013). It is challenging to assess because of the limited knowledge of the range of relevant infectious (and noninfectious) hazards that may affect a conservation translocation (Sainsbury & Carraro, 2023). Even where evidence is available, translocations inevitably require extrapolating that evidence in space and time. Use of DRA will thus always require expert judgment, which will in turn involve substantial uncertainty. Recognizing this, current approaches should adopt best practices for elicitation and treatment of such judgments. We recommend that judgments be elicited from multiple experts through a structured process such as the IDEA protocol, expressed as quantitative measures on natural scales rather than verbal descriptions, and presented and compared across management alternatives, and that uncertainty and noise among experts be expressed transparently and aggregated appropriately. These improvements to qDRA help reveal uncertainty and noise and provide decision makers with direct information about risk mitigation. Our modifications require more effort in the sense of engaging multiple experts but also have the potential to streamline the process via structured group assessments that do not require many months of independent work by single risk assessors. Our recommendations provide an honest reflection of expert judgment needed for making better conservation translocation decisions.

## Supporting information



Supporting Information
