# Supplementary material for: Improving the use of expert opinion in disease risk analysis for conservation translocations
Source: Conserv Biol. 2026 Apr 24;40(4):e70292. doi: 10.1111/cobi.70292 (PMC13392744; doi:10.1111/cobi.70292)

**Supplementary Information**

**Appendix S1.** List of terrestrial vertebrate species and potential vector species on Palmyra Atoll.

| **Taxon** | **Family** | **Common Name** | **Binomial Name** |
| --- | --- | --- | --- |
| **List of terrestrial vertebrate species** | | | |
| Bird | Charadriidae | Pacific Golden Plover | *Pluvias fulva* |
| Bird | Scolopacidae | Ruddy Turnstone | *Arenaria interpres* |
| Bird | Scolopacidae | Sanderling | *Calidris alba* |
| Bird | Scolopacidae | Bristle-thighed Curlew | *Numenius tahitiensis* |
| Bird | Scolopacidae | Wandering Tattler | *Tringa incana* |
| Bird | Sternidae | Black Noddy | *Anous minutus* |
| Bird | Sternidae | Brown Noddy | *Anous stolidus* |
| Bird | Sternidae | White Tern | *Gygis alba* |
| Bird | Sternidae | Sooty Tern | *Onchoprion fuscatus* |
| Bird | Phaethontidae | White-tailed Tropic Bird | *Phaethon lepturus* |
| Bird | Phaethontidae | Red-tailed Tropic Bird | *Phaethon rubricauda* |
| Bird | Fregatidae | Great Frigate Bird | *Frigata minor* |
| Bird | Sulidae | Masked Booby | *Sula dactylatra* |
| Bird | Sulidae | Brown Booby | *Sula leucogaster* |
| Bird | Sulidae | Red-footed Booby | *Sula sula* |
| Reptile | Gekkonidae | Sexual Mourning Gecko | *Lepidodactylus sp. nov.* |
| Reptile | Gekkonidae | Mourning Gecko | *Lepidodactylus lugubris* |
| **List of potential vector species** | | | |
| Arthropod | Hippoboscidae | Louse Fly | *Ornithoica vicina* |
| Arthropod | Culicidae | Asian Tiger Mosquito | *Aedes albopictus* |
| Arthropod | Culicidae | Southern House Mosquito | *Culex quinquefasciatus* |
| Arthropod | Ceratopogonidae | Biting Midge | *Ceratopogonidae sp.* |
| Arthropod | Sarcophagidae | Wretched flesh fly | *Parasarcophaga misera* |
| Arthropod | Sarcophagidae | Flesh Fly | *Parasarcophaga sp.* |
| Arthropod | Sarcophagidae | Peregrin Flech Fly | *Sarcophaga peregrina* |
| Annelid | Haemadipsidae | Terrestrial Leech | *Hirudinea sp.* |


**Appendix S2.**

Detailed risk assessments for seven pathogen hazards.

**Hazard 1: West Nile Virus (WNV) (Source Hazard)**

**Justification of hazard**

West Nile Virus (WNV) is a positive-stranded RNA mosquito-transmitted virus belonging to the *Flavivirus* genus in the *Flaviviridae* family and is responsible for the zoonotic disease West Nile Disease (also known as West Nile Fever). WNV is maintained in an enzootic bird-mosquito cycle, with birds acting as reservoirs and amplifying hosts. Mosquitoes, mainly of the *Culex* genus, become infected by feeding on viraemic birds and can then potentially transmit the virus to every vertebrate they feed on (Reid et al, 2012).

There are different strains of WNV grouped into seven separate genetic lineages (Mackenzie & Williams, 2009), with lineage 1 and lineage 2 representing the major ones. Lineage 1 is widespread and includes isolates from Africa, Europe, Asia, America, the Middle east, India, and Australia (Lanciotti et al, 2002; Charrel et al, 2003); lineage 2 had originally been mostly restricted to sub-Saharan Africa and Madagascar, however it is now endemic in several countries of central and southern Europe (Valiakos et al, 2011; Wodak et al, 2011; Savini et al, 2012; Bakonyi et al, 2013).

WNV was firstly introduced into North America, New York, in 1999 (Lanciotti et al, 1999), becoming endemic within ten years of its introduction in this continent (De Filette et al, 2012). It has so far been detected in over 300 species of birds causing thousands of deaths (CDC Database).

There is no evidence of WNV being present on Palmyra Atoll. However, it is important to note that competent vectors (*Culex* spp.) are known to be present. WNV is therefore considered a source hazard to the sihek conservation translocation project

**Situation in sihek**

To date, WNV-associated disease and mortality in captive sihek have been reported in three birds (one was euthanised and WNV positivity confirmed via PCR).

**Release assessment**

Birds’ susceptibility to WNV infection and competency to amplify and transmit the virus varies depending on the species, and passerines have been identified as the most competent order of birds (Komar et al, 2003). Within passerines, species belonging to the Corvidae family are the most susceptible ones, showing extremely high titers of viraemia and mortality (Komar et al, 2003), thus suggesting their primary role (together with house sparrows) in WNV amplification. Interestingly, Kilpatrick and colleagues’ results (2006) propose that the American robin (*Turdus migratorius*) may be the most important amplification host (main reservoir of the virus) in urban and residential areas in the USA. In endemic areas, infection in wild birds has a seasonal pattern, usually starting in spring and early summer with mortality peaks from midsummer to early autumn.

The primary route of WNV transmission is through the bite of infected mosquitoes, although Komar and colleagues (2003) have experimentally demonstrated that in certain avian species virus spread may occur by oral route (through ingestion of contaminated food/water) and via close contact with other infected birds (in the absence of mosquito-borne transmission). The mode of this ‘close contact transmission’ remains unknown and so does its occurrence in the wild.

The WNV strain introduced in North America is more virulent compared to the strains circulating in Europe and continues to circulate and expand its distribution, being found in all 48 contiguous states (not in Alaska). The extremely high viremias produced in species of the Corvidae family is responsible not only for significant avian mortality but also for an increased transmission potential to vector mosquito species (intense viral circulation).

**Exposure Assessment**

In birds, the exact pathogenesis (i.e., the mechanism and sites of viral replication within the host) of WNV infection is yet to be fully understood but the virus can generally be detected in the blood within one day after the mosquito bite. Depending on the susceptibility of the species, viremia can peak as early as 2-3 days post infection or 4-6 days post infection, and the virus can be isolated up to 10 days post infection (in Gamino & Höefle, 2013). The invasion of the CNS and/or other major organs determines the onset of clinical disease, with symptoms usually appearing 5 days post experimental infections although asymptomatic infection have also been reported (in Gamino & Höefle, 2013).

For other susceptible bird species to become infected at the destination site, competent vectors are required and sufficiently high titers of viraemia in reintroduced sihek are needed to infect such competent vectors. The southern house mosquito (*Culex quinquefasciatus*) was introduced to Palmyra Atoll during World War II and the suitable wet tropical climate has resulted in abundant mosquito populations (Lafferty et al, 2018). Avian host species able to develop viremia greater than 10^6^ PFU ml^-1^ are considered competent reservoirs. The competency of sihek to amplify and transmit the virus, thus acting as a reservoir for WNV, is currently unknown.

**Consequence Assessment**

Both viral and host factors play an important role in the outcome of avian WNV infections. The reason behind the high rates of clinical disease and extensive mortality of North American wild bird species, which has followed WNV introduction in New York in 1999, is probably the increased virulence of the introduced virus strain and the naivety of avian species. Cross-protection from other antigenically related flaviviruses may reduce the likelihood of disease outbreaks. Concurrent diseases, the influence of hormonal factors and stress, as well as age, are factors that can affect the immune system of the host and therefore influence its capacity to clear the virus.

**Hazard 2: Avian Poxvirus (APV) (Source Hazard)**

**Justification of hazard**

Avian poxvirus (APVs; genus *Avipoxvirus*, family *Poxviridae*) are responsible for avian pox, probably the oldest recognised avian viral disease with a worldwide distribution (Bolte, Meurer, & Kaleta, 1999; van Riper III & Forrester, 2007). Within the *Avipoxvirus* genus there are currently ten recognized species, named after the respective taxonomic avian group of origin: canarypox, fowlpox, juncopox, mynahpox, pigeonpox, psittacinepox, quailpox, sparrowpox, starlingpox, and turkeypox (ICTV, 2018). However, the exact number of existing APV species, strains and variants is unknown and new isolates continue to be identified from a wide variety of avian species (Gyuranecz et al, 2013). APVs occur worldwide except in the Arctic and some remote oceanic islands (van Riper III & Forrester, 2007; Shearn-Bochsler et al, 2008), generally at higher prevalence in temperate and warmer areas of the globe where conditions are suitable for abundant and active vector populations. For instance, in North America APVs appear to be more prevalent in the moister and warmer south eastern regions (van Riper III & Forrester, 2007). The presence of APVs on Palmyra Atoll is currently unknown, but as far as the authors are aware no infection/disease cases have been reported to date. Most birds, if adequately exposed, are susceptible to one or more APV strains and to date natural infection has been recorded in over 270 avian species (van Riper III & Forrester, 2007). Classically, APVs are relatively host-species or host-order specific, however some APVs have a broader host range and are able to infect several avian species.

APV infections are responsible for significant economic losses in domestic poultry but effects can be severe on wild bird species too, particularly on small, endangered island bird populations. APV introduction has been disastrous for endemic birds on remote islands (e.g., Hawaii (van Riper III, van Riper & Hansen, 2002; Tripathy et al, 2000); Galápagos (Thiel et al, 2005); Canary Islands (Smits et al, 2005); Falkland Islands (Munro, 2006) causing epizootics characterized by high morbidity and high mortality that have resulted in population decline of native bird species.

APV strains found at the source site may not be present at destination and would therefore constitute a source hazard for the sihek translocation to Palmyra Atoll. Some authors suggest that APV infections may remain latent for long periods of time, and non-specific stressors may then allow the virus to become reactivated triggering clinical disease in the host and spreading to other avian hosts (Garner, 1989; Forbes & Simpson, 1997); latency has however not been confirmed in any avian species yet (Deem, Heard, & Fox, 1997). Since translocation is likely to act as a stressor to the sihek, APV could also act as a potential carrier hazard.

**Situation in sihek**

Infection and disease have sporadically been reported in captive sihek (N=4) as well as in one other Alcedinidae species, all held at the same institution. In two cases molecular identification confirmed that the virus involved was identical to that found in affected condors housed at the same institution.

**Release assessment**

APV is unable to penetrate intact skin or epithelial surfaces and must enter the host through skin abrasions (insect bites, trauma). Mosquitoes, especially *Culex* and *Aedes* spp., and other blood-sucking arthropod vectors (mites, midges and/or flies) play a major role in the transmission of APV, mechanically transferring the virus from infected to susceptible birds (van Riper III & Forrester, 2007). Mosquitoes acquire the virus when feeding from an infected bird and the virus may remain up to eight weeks in the vectors’ salivary glands, with no further replication, during which period it can be transmitted to other susceptible birds (Bourne, Duff, & Vikøren, 2012). Suitable vectors are known to be present at the source sites. Viral transmission may also occur directly by contact between infected and susceptible birds or indirectly by contact with contaminated surfaces (perches, feeders, water bowls, nests, crates) provided the skin of the host has been compromised. Aerosol transmission, although rare, may also occur from viruses being carried along with dust, dried scabs, or other particles, particularly in confined situations such as aviaries. When present in an aerosol, APV can reach and penetrate apparently intact mucous membranes. Likely due to their very large size, APVs are extremely resistant even in extreme environmental conditions and can withstand desiccation thus surviving on perches and dried scabs for several months or years (Tripathy, 1993).

Host density, number of vectors and host susceptibility are the most important factors influencing avian pox epidemiology (van Riper III & Forrester, 2007). Viral transmission is enhanced with increasing host and vector densities and, in temperate regions, the prevalence of infection is higher during the summer and early autumn. However, in continental areas where avian pox is endemic and the virus and its hosts have had a long co-evolutionary history, the prevalence of lesions on wild birds is quite low, ranging between 0.5% and 1.5% (van Riper III & Forrester, 2007).

**Consequence assessment**

The pathogenesis of APV infection differs depending on the viral strain involved. Non-pathogenic and/or low pathogenic strains remain localized at the site of entry, causing localised lesions, while pathogenic strains are responsible for an initial viremia followed by distribution of the virus to the liver and bone marrow with further replication, secondary viremia, and systemic disease. Viral strain and host species are also responsible for the length of the incubation period which spans from a few days in domesticated avian species to several months in some wild bird species (Bourne, Duff, & Vikøren, 2012). Sihek that are exposed to and become infected with APV at the source site will carry the virus to Palmyra Atoll. Here, competent vectors are present and year-round active given the tropical climate.

**Hazard 3: Aspergillus fumigatus (Carrier Hazard)**

**Justification of hazard**

*Aspergillus fumigatus* is a naturally occurring cosmopolitan fungus which causes the disease aspergillosis (Converse, 2007a). Its spores can be distributed by global wind currents (Smith et al, 1996) and, with the exception of Antarctica, it has a worldwide distribution (Converse, 2007a). It is ubiquitous, found in soil, decomposing organic matter, litter, agricultural waste and moist, warm (23^o^ to 26^o^C) environments promote both fungal growth and sporulation (Converse, 2007a). Nevertheless, *A. fumigatus* may be less abundant in mature (undisturbed) forests compared to modified environments (Perrott & Armstrong, 2011). We assume it is present on Palmyra Atoll.

Aspergillosis could develop because either i) the initial dose of inhaled spores is so high that it exceeds the bird’s natural resistance; or ii) birds may carry the fungal spores in their lungs and air sacs until immune suppression, possibly as a consequence of stress, triggers clinical disease (Friend & Trainer, 1969 as cited in Converse, 2007a). The translocation process might act as a stressor to the sihek and as a consequence aspergillosis could occur. *A. fumigatus* can therefore be categorized as a carrier hazard, although it should be noted that it is more of an opportunistic pathogen rather than a commensal infectious agent.

**Situation in sihek**

Both infection and disease have been recorded in captive sihek as well as in other Coraciiformes held at different institutions.

**Release assessment**

Inhalation of airborne spores is the main transmission route (Converse, 2007a) and, because of their small size, the spores are not trapped completely in the nasal cavity or trachea and some are able to reach the lungs and air sacs (Fedde, 1998). The aerobic environment of the respiratory tract allows spores to germinate, producing fungal hyphae which induce a strong inflammatory cell response that results in the formation of typical lesions (firm, pale nodules within the lung and/or pale, rubbery plaques on the air sacs (Converse, 2007a). Infection can spread to other internal organs via fungal dissemination through the vascular system and pneumatized bones.

In birds, another plausible transmission route is through fungal spores penetrating the eggshell, which may result in either embryo mortality or chicks hatching already infected or with signs of disease (Olsen, Nicolich, & Hoffman, 1990 as cited in Converse, 2007a).

*Aspergillus fumigatus* is ubiquitous and, depending on spore load in the environment, exposure might happen at different points along the translocation pathway: i) spores could penetrate eggshells; ii) sihek chicks, if not already exposed through eggshells, could be exposed once hatched via inhalation of the spores. Humidity, a warm environment, poor ventilation (Phalen, 2000; Tell, 2005) and poor sanitation (Oglesbee, 1997) are factors capable of increasing the number of spores in the air and spores are also extremely persistent in the environment. Once exposed, sihek are likely to become infected because the species is known to be susceptible. It should also be noted that juvenile birds are particularly susceptible to infection and disease (Jansson, 2012). The probability of disease is increased if the immune status of sihek is compromised through stressors associated with the translocation process.

**Exposure assessment**

Sihek exposed to and infected with *A. fumigatus* at any stage of the translocation pathway are likely to retain the infection. Aspergillosis is an infectious but not contagious fungal disease, which spreads neither by horizontal (bird to bird) nor by vertical (dam to egg) transmission (Kearns, 2014). The likelihood that a fungal infection will infect and disseminate amongst sihek and other birds at the destination site is negligible. However, frequently more than one bird in a group is affected as a result of exposure to the same stressors or other environmental conditions.

**Consequence assessment**

An increased concentration of spores in the environment and a compromised immune response in individual animals can result in unsuccessful elimination of the infection and may predispose a bird to aspergillosis (Beernaert et al, 2010). Numerous factors compromising a bird’s immunity can also make individuals more susceptible to infection and disease. Examples of factors increasing the risk of developing aspergillosis, once infected, include overcrowding (McMillan and Petrak, 1989), shipping (Tshai et al, 1992), quarantine or capture of wild birds (Abrams et al, 2001), metabolic bone disease (Vanderheyden, 1993) and traumatic injuries (Xavier, 2008). Avian aspergillosis is often classified as acute or chronic. Acute aspergillosis primarily occurs in young and recently translocated birds (Woodford and Rossiter, 1994) and is thought to be the result of inhaling an overwhelming number of spores (Vanderheyden, 1993). Chronic aspergillosis is more likely to occur in older birds that have been in captivity (Locke, 1987) and is generally associated with immune suppression (Vanderheyden, 1993).

Although aspergillosis is predominantly a disease of the respiratory tract, other organs can be involved leading to a variety of clinical manifestations. Breathing difficulties (dyspnoea, gasping, polypnoea), sleepiness (somnolence) and other signs of nervous system involvement, inappetence, emaciation and increased thirst may be seen (Kearns, 2014).

**Hazard 4:** ***Chlamydophila psittaci* (Carrier Hazard)**

**Justification of hazard**

Chlamydiae are Gram-negative, obligate intracellular bacteria and those affecting avian species belong to the order *Chlamydiales*, genus *Chlamydophila*, species *C. psittaci* which contains all six avian serovars (A-F) known to date (Andersen & Franson, 2007). *C. psittaci* has a worldwide distribution and it has been isolated, or antibodies have been serologically detected, in 467 avian species belonging to 30 different orders (Kaleta & Tadai, 2003). We assume it is present on Palmyra Atoll.

*C. psittaci* is usually highly prevalent in Psittaciformes and Columbiformes but Anseriformes, Charadriiformes, Falconiformes, Passeriformes, Procellariformes, and Strigiformes are also important natural carriers of the bacterium (Aaziz et al, 2015). Considering the wide range of hosts, all wild birds are assumed to be susceptible to infection and disease. In a recent study by Sukon and colleagues (2021) the global prevalence of chlamydial infections in birds was 19.5 % (95 % CI, 16.3 % - 23.1 %), with no significant differences among continents (in North America the prevalence was 21.7 % (95 % CI, 12.1 % - 35.9 %).

Current knowledge indicates that chlamydial strains are highly host specific, producing a mild to moderate disease in natural hosts, with persistent infection after recovery. Still, some strains are also capable of being transmitted to and infect other non-natural hosts in which, depending on the virulence, effects might span from no infection to severe disease and possible death; however, persistence of infection does not occur in non-natural hosts (Andersen & Franson, 2007). Young birds are usually considered more susceptible than adults to both infection and disease. Stress factors can result in increased susceptibility to infection and also trigger disease in those persistently infected birds which will also resume shedding of the organism.

Considering that translocation can be a stressful process and that sihek will be moved at a young age, *C. psittaci* has been categorised as and is here assessed as a carrier hazard.

**Situation in sihek**

Avian chlamydiosis has been diagnosed at least twice in captive sihek (information retrieved from grey literature), however no infection/disease cases were found during the survey of data provided by several sihek holding institutions for this DRA. Both cases recorded in sihek were asymptomatic and diagnosis was only made during post-mortem examination.

**Release assessment**

Chlamydiae are shed by infected birds, sometime for prolonged periods, in their faeces, nasal discharges, eye secretions. *C. psittaci* is therefore mainly horizontally transmitted, generally through inhalation of infectious aerosolized respiratory secretions/dried faeces or ingestion of contaminated dried dust particles. *C. psittaci* is susceptible to most disinfectants and detergents, as well heat (Pal, 2017).

Although not particularly efficient and probably relatively unusual, vertical transmission through the egg has been reported in few species, namely turkeys, chickens, wild geese, and ducks (Lehnert, 1962; Illner, 1962; Wilt, Kordova, & Wilt, 1972; Wittenbrink, Mrozek & Bisping, 1993; and Lublin et al., 1996 as cited in Andersen & Franson, 2007) and should therefore be considered. Another possible but rare route of transmission is through mechanical vectors, ectoparasites (Andersen & Franson, 2007).

If exposed, sihek are likely to become infected because the species is known to be susceptible. The probability of disease is increased if the immune status of sihek is compromised through stressors associated with the translocation process.

**Exposure assessment**

Chlamydiae are known to survive in a commensal relationship in the gastrointestinal tract of infected hosts, with precipitation of disease under stressful circumstances (Speck & Duff, 2012). Stress associated with the translocation (e.g., handling, prolonged transport, overcrowding) may therefore trigger disease in those infected sihek. Stress can also exacerbate shedding of chlamydiae in which case dissemination of the infection amongst the group of translocated sihek and/or other susceptible species at destination could be possible. The survival of chlamidiae in the environment is facilitated by moist conditions (Brand, 1989, as cited in Andersen & Franson, 2007).

**Consequence assessment**

In domestic poultry and pet birds, chlamydiosis can be acute, subacute, or chronic. Non-specific clinical signs (lethargy, anorexia, ruffled feathers, weight loss, failure to thrive) are commonly reported. Ocular discharge and localized conjunctivitis, dyspnoea, pneumonia and airsacculitis, enteritis and diarrhoea, might be observed. All these findings are non-specific and non-diagnostic in themselves. Tetracyclines, in particular doxycycline, are usually used in treatment of sick birds.

**Hazard 5:** ***Trichomonas gallinae* (Source Hazard)**

**Justification of hazard**

*Trichomonas gallinae* is a cosmopolitan, flagellate protozoa belonging to the family *Trichomonadidae* and is commonly found in birds, for which it represents the only trichomonad species with a non-ambiguous, proven pathogenic potential (Bondurant & Honigberg, 1994). It is responsible for avian trichomonosis, a disease reported from several parts of the world and of particular importance especially in Columbiformes and raptors (Forrester & Foster, 2008). Its distribution closely follows that of its main host, the rock pigeon (*Columba livia*) which is considered responsible for the worldwide protozoan distribution, still other species of Columbiformes like wood pigeons (*Columba palumbus*) and doves (e.g., collared dove, *Streptotelia decaocto*) are similarly important hosts of the protozoa (Amin et al, 2014). Other hosts include Galliformes, Psittaciformes, Passeriformes, Gruiformes, and Anseriformes (Forrester & Foster, 2008; Amin et al, 2014). Virulence and pathogenicity greatly vary among different strains of *T. gallinae*, with most strains being non-pathogenic or moderately pathogenic and some others being virulent.

**Situation in sihek**

To date and as far as the authors are aware, infection and disease have not been recorded in captive sihek and/or other captive *Coraciiformes*, nevertheless post-mortem examination of a 4 days old sihek chick that died after failing to thrive revealed a necrotizing ventriculitis, with accumulation of necrotic cell debris and abundant pyriform organisms, most compatible with trichomonad species. However, final diagnosis of trichomonosis was not possible and remains hypothetical because staining attempts by Periodic-acid Schiff (PAS) technique of the organism failed. It should be noted, though, that the use of PAS to detect trichomonads in tissues has proven a limited sensitivity (Amin et al, 2011).

**Release assessment**

*T. gallinae* has a direct life cycle that does not involve intermediate or paratenic hosts. The main route of transmission is by direct contact with the saliva of an infected bird. In Columbiformes, this can happen when parents feed their chicks (via pigeon milk regurgitation), while cross-feeding or billing during courtship (Forrester & Foster, 2008). Contaminated water or food may also act as sources of infection (Gómez-Muňoz et al, 2018) and the protozoa has proven the ability to live up to several hours in water depending on the salinity and for at least 5 days in moist grains (Kocan, 1969).

*T. gallinae* is a fragile protozoan which displays only a low tenacity in the environment being rapidly killed by desiccation. Although no true resistant cystic form of the protozoa is known, cyst-like stages (pseudocysts) have been demonstrated but their role in the parasite’s life cycle and whether they may extend survival time outside the host remain to be determined (Tasca & De Carli, 2003; Forrester & Foster, 2008).

**Exposure assessment**

Upon ‘entry’ into a host, *T. gallinae* is mainly found in the upper digestive tract (mouth, pharynx, oesophagus, and crop) and only rarely posterior to the proventriculus. Excretion of the protozoa via droppings is therefore very limited (Amin et al, 2014). Sihek that are exposed to and become infected with *T. gallinae* at the source site (assuming their susceptibility to infection) will carry the protozoa to Palmyra Atoll. Here other avian species might become exposed to the protozoa by using the same feeding and watering areas as infected sihek, however their susceptibility to infection once exposed is not known.

**Consequence assessment**

Severity of disease depends on the host susceptibility as well as the type of strain and its pathogenic potential. While infections with moderately pathogenic strains result in mild symptoms like sialorrhea (excessive salivation) and mild inflammation of the oral cavity and throat, infections with more pathogenic strains result in more severe caseous lesions in the mouth, throat, and crop as well as invasion of internal organs in some cases. In most severe cases, death can occur as early as 4 days post infection (Forrester & Foster, 2008).

**Hazard 6:** ***Isospora* spp. (Carrier Hazard)**

**Justification of hazard**

*Isospora* spp. are protozoan parasites, also known as coccidian parasites, belonging to the Apicomplexa phylum along with members of the genera *Eimeria*, *Cryptosporidium*, *Toxoplasma* and members of the suborder Eimeriorina (Gosbell et al, 2020). They have a worldwide distribution and are most described with a high prevalence (up to 90%) in passerine species from several countries (Gosbell et al, 2020). They usually have a direct life cycle, completing their development in the intestinal epithelium of avian hosts. Infections with enteric (intestinal) species of *Isospora* do not usually cause disease, however if the host is naïve to the parasite, or subjected to stressors, disease may develop, and it is referred to as coccidiosis. Besides these enteric species, there are some *Isospora* spp. that have a more complex life cycle involving an extraintestinal asexual life-stage, in monocytes, and they are responsible for a disease called systemic isosporosis (formerly known as atoxoplasmosis). Systemic isosporosis represents an important protozoal disease of passerine birds, especially those managed in captivity (e.g., the Bali Mynah *Leucopsar rothschildi*, Greiner, 2008)

Morbidity and mortality linked to systemic isosporosis have been shown to be highest in juvenile birds (canaries, aged 2 to 9-months), whereas adults are generally asymptomatic and most likely to develop systemic disease under stressful circumstances and/or upon ingestion of high doses of infective oocysts (Adkesson, Zdziarski, & Little, 2005). Findings by Gill and Paperna (2008) as cited in Gosbell et al. (2020), also suggest that wild free-living birds can usually coexist with systemic *Isospora* spp. infection only to succumb to disease following stressful situations (e.g., capture).

Since the proposed translocation pathway involves the movement of young sihek chicks, and considering the translocation procedure itself may act as a stressor, disease may occur.

**Situation in sihek**

Unidentified species of coccidia, sometimes indicated as *Isospora* spp., have been reported by several holding institutions during routine faecal screening of captive sihek. Given it is not possible to distinguish oocysts of enteric species of *Isospora* from those of systemic species of *Isospora* by microscopic examination of faeces (both oocysts containing two sporocysts, each with four sporozoites), a lot of uncertainty remains regarding what species of the parasite, enteric vs systemic, are mostly found in the sihek captive population. No cases of coccidiosis within the captive sihek population were found during our review of the necropsy data provided by those institutions which contributed to this DRA process. However, the necropsy of a two and half months old, parent-reared chick that died at one holding institution revealed lesions consistent with systemic *Isospora* spp. infection (diagnosis based on histopathology).

**Release assessment**

Regardless of whether the parasite is an enteric or a systemic species of *Isospora*, transmission occurs through ingestion of sporulated, infective, oocysts (faecal-oral route). Unsporulated, non-infective, oocysts enter the environment in the faeces of an infected host. Under favourable conditions of oxygen, humidity and temperature, oocysts sporulate and become infective within a few days (Greiner, 2008). A sihek must then ingest these oocysts when feeding or drinking and the parasite can then proliferate and undergo sexual maturation in the epithelial cells of the small intestine as well as a systemic asexual phase in circulating monocytes (the latest phase only applying to systemic *Isospora* spp.). During such extraintestinal phase, the parasite is intracellularly disseminated through the blood stream to multiple organs, thus leading to a systemic manifestation of the disease.

It is worth noting that while some authors (Mohr, Betson, & Quintard, 2017) have suggested vertical transmission of the parasite (across the oviduct) as a possible route of infection, results from a previous study were in disagreement with such a theory (Adkesson, Zdziarski, & Little, 2005). In both studies, however, additional research has been advocated before any conclusions could be made. To date vertical transmission has yet to be confirmed.

Coccidian oocysts in the environment are practically ubiquitous in highly populated confined areas (e.g., aviaries) and mechanical transfer (through rodent vermin, flying insects, other invertebrate pests, humans, contaminated food, old litter) can also occur. Moreover, oocysts have a thick wall which protects them from heat, cold, and even most common disinfectants, enabling them to survive and remain infective in the environment for long periods of time, months to years (Ryley, 1973). Nestlings may be more prone to acquire a large build-up of infective oocysts within a short period of time considering increased shedding by adults has been seen during egg laying, brooding, and while feeding chicks in the nest (Greiner, 2008).

**Exposure assessment**

Sihek nestlings that become infected with *Isospora* spp. at the source site will carry the parasite to Palmyra Atoll. Generally, enteric species of *Isospora* are highly species-specific, but the level of host specificity for systemic *Isospora* spp. is currently unknown. However, evidence for some host specificity as well as some indication of possible transmission among unrelated host species have been shown (Schrenzel et al, 2001 as cited in Greiner, 2008).

**Consequence assessment**

Clinical signs of coccidiosis include diarrhoea, fever, inappetence, weight loss, emaciation, and in extreme cases, death. Clinical signs associated with systemic isosporosis might mimic those of coccidiosis. However, in the absence of stressors, most infections are subclinical. In general, clinical healthy, adult birds can be sources of infection to young susceptible nestlings. The immature immune system of chicks makes them more susceptible to develop clinical coccidiosis which can also be precipitated by stress.

**Hazard 7:** ***Mycobacterium* sp. (Carrier Hazard)**

**Justification of hazard**

Mycobacteria (order Actinomycetales, family Mycobacteriaceae, genus *Mycobacterium*) are a group of intracellular, rod-shaped, acid-fast Gram-positive, non-spore-forming, aerobic bacteria which are ubiquitous, existing as saprophytic organisms in soil and water. They are highly persistent in the environment due to their lipid-rich waxy cell wall that protects them from desiccation, ultraviolet light and cold temperature (Converse, 2007b). Mycobacteria are responsible for mycobacteriosis, a contagious, chronic, slow-developing disease. Mycobacteriosis occurs worldwide in domestic, captive, and wild birds, although is more commonly diagnosed in the north temperate zones (Riggs, 2012). The disease is relatively uncommon in captive birds when these are held individually or in small groups, as found in a survey of 23,960 necropsies of captive birds in the U.S. with mycobacteria reported in only 1.3% of the cases (Riggs, 2012). Some studies have reported an even lower disease prevalence in free-ranging wild birds, for example Franson and Friends (1999) as cited in Riggs (2012) have found a disease prevalence of less than 1% in North American wild birds which is comparable to a 0.7% prevalence in a study of 12,000 birds in the Netherlands. Also, a 0.3% infection rate has been found in a review of 3,000 U.S. wild waterfowl necropsies (Franson & Friends, 1999 as cited in Riggs, 2012). A noteworthy exception is that of a 39% disease prevalence found in a free-ranging U.S. whooping crane (*Grus americana*) population, likely due to environmental conditions favouring infection and pathogen dissemination (Riggs, 2012). Mycobacteriosis is more common in high density captive conditions, although prevalence still varies as shown by necropsy reviews from three U.S. zoological institutions that reported 1.2%, 4%, and 24.5% disease prevalence (Beehler, 1990; Converse, 2007b; Witte et al, 2007 as cited in Riggs, 2012). In birds, disease is generally caused by infection with *M. avium* complex (MAC, it includes two main species: *M. avium* and *M. intracellulare*) and *M. genavense*.

Stress may increase the susceptibility to disease in birds.

**Situation in sihek**

Severe, disseminated mycobacteriosis has been identified as an important cause of mortality in the captive sihek population, being recorded across several holding institutions and mainly caused by *M. avium* complex (MAC) and *M. genavense*. In addition to these, atypical mycobacteriosis caused by *M. simiae* complex has also been documented (Travis, Junge, & Terrell, 2007) at different holding institutions.

**Release assessment**

Transmission can occur in different ways, but the main route is by ingestion of contaminated food or water (faecal/oral route). Inhalation of bacilli from aerosols, either directly from infected birds with lesions in the respiratory tract or from a contaminated environment, is also an important route of transmission, especially in captivity. Potential vertical transmission has been documented in domestic poultry but not in free-living birds, and it is thought to be relatively uncommon (Converse, 2007b; Cromie, 2012). However, young chicks could be easily exposed and become infected from faecal contamination of the eggs, nest, and environment (Cromie, 2012). Another suggested, less likely, mode of transmission is through mechanical transmission by arthropods (e.g., ticks, cockroaches, flies) and small vertebrates (e.g., wild rodents) (Converse, 2007b; Cromie, 2012).

**Exposure assessment**

Sihek nestlings that become infected with *Mycobacterium* spp. at the source will carry the parasite to Palmyra Atoll. *Mycobacterium* spp. are however assumed to be already present at destination considering their ubiquity in the environment. All avian species are considered susceptible to infection with *Mycobacterium* spp.

**Consequence assessment**

Adult birds are more commonly affected because of the long incubation period and accumulated risk of exposure (mainly through faecal-oral route). The role of stress on recently translocated sihek should always be considered though, as it could have an impact on latent infections.

Mycobacteriosis typically occurs sporadically in wild free-living birds and usually affects individuals rather than producing group mortality events (Converse, 2007b).

**References listed**

Aaziz, R., Gourlay, P., Vorimore, F., Sachse, K., Siarkou, V.I., and Laroucau, K. 2015. Chlamydiaceae in North Atlantic seabirds admitted to a wildlife rescue center in western France. Applied and Environmental Microbiology, 81(14): 4581-4590.

Adkesson, M.J., Zdziarski, J.M., and Little, S.E. 2005. Atoxoplasmosis in tanagers. Journal of Zoo and Wildlife Medicine, 36: 265-272.

Amin, A., Bilic, I., Liebhart, D. and Hess, M. 2014. Trichomonads in birds – A review. Parasitology, 141: 733-747. Available from: doi:10.1017/S0031182013002096.

Amin, A., Liebhart, D., Weissenbock, H. and Hess, M. 2011. Experimental infection of turkeys and chickens with a clonal strain of *Tetratrichomonas gallinarum* induces a latent infection in the absence of clinical signs and lesions. Journal of Comparative Pathology, 144: 55-62. Available from: doi: 10.1016/j.jcpa.2010.06.002.

Andersen, A.A., and Franson, J.C. 2007. Avian Chlamydiosis. In: Thomas, N.J., Hunter, D.B., & Atkinson, C.T. (Eds), *Infectious Diseases of Wild Birds* (pp. 303-316). Blackwell Publishing Ltd., UK.

Abrams, G.A., Paul-Murphy, J., Ramer, J.C., and Murphy, C.J. 2001. Aspergillus blepharitis and dermatitis in a peregrine falcon-gyrfalcon hybrid (*Falco peregrinus* x *Falco rusticolus*). Journal of Avian Medicine and Surgery, 15: 114-120.

Bakonyi, T., Ferenczi, E., Erdélyi, K., Kutasi, O., Csörgő, T., Seidel, B., Weissenböck, H., Brugger, K., Bán, E., and Nowotny, N. 2013. Explosive spread of a neuroinvasive lineage 2 West Nile virus in Central Europe, 2008/2009. Veterinary Microbiology, 165: 61-70.

Beehler, B.A. 1990. Management of *Mycobacterium avium* in a mixed species aviary. In: Proceedings of the American Association of Zoo Veterinarians, pp. 125-129. Yulee. FL.

Beernaert, L., Pasmans, F., van Waeyenberghe, L., Haesebrouck, F., and Martel, A. 2010. Aspergillus infections in birds: a review. Avian Pathology, 39(5): 325-331.

Bolte, A.L., Meurer, J., and Kaleta, E.F. 1999. Avian host spectrum of avipoxviruses. Avian Pathology, 28(5): 415-432.

Bondurant, R.H., and Honigberg, B.M. 1994. Trichomonads of veterinary importance. In: Kreier, J.P. (ed.) *Parasitic Protozoa*. Academic Press, New York, NY, USA. Pp. 111-188.

Bourne, D., Duff, J.P., and Vikøren, T. 2012. Poxvirus infection. In: Gavier-Widen, G., Duff, P.D. & Meredith, A. (Eds), *Infectious Diseases of Wild Mammals and Birds in Europe* (First edition pp. 191-209). Blackwell Publishing Ltd., UK

CDC Database. https://www.cdc.gov/west-nile-virus/about/index.html

Charrel, R.N., Brault, A.C., Gallian, P., Lemasson, J.-J., Murgue, B., Murri, S., Pastorino, B., Zeller, H., de Chesse, R., de Micco, P., and Lamballerie, X. 2003. Evolutionary relationship between Old World West Nile virus strains evidence for viral gene flow between Africa, the Middle East, and Europe. Virology, 315: 381-388.

Converse, K.A. 2007a. Aspergillosis. In: Thomas, N.J., Hunter, D.B., & Atkinson, C.T. (Eds), *Infectious Diseases of Wild Birds*. Blackwell Publishing Ltd., UK. Pp: 360-374.

Converse, K.A. 2007b. Avian Tuberculosis. In: Thomas, N.J., Hunter, D.B., & Atkinson, C.T. (Eds), *Infectious Diseases of Wild Birds*. Blackwell Publishing Ltd, Oxford, UK. Pp: 289-302.

Cromie, R. 2012. Avian Tuberculosis. In: Gavier-Widen, G., Duff, P.D. & Meredith, A. (Eds), *Infectious Diseases of Wild Mammals and Birds in Europe* (First edition pp. 274-281). Blackwell Publishing Ltd., UK

Deem, S.L., Heard, D.J., and Fox, J.H. 1997. Avian pox in eastern screech owls and barred owls from Florida. Journal of Wildlife Diseases, 33(2): 323-327.

De Filette, M., Ulbert, S., Diamond, M., and Sanders, N.N. 2012. Recent progress in West Nile virus diagnosis and vaccination. Veterinary Research, 43:16.

Fedde, M.R. 1998. Relationship of structure and function of the avian respiratory system to disease susceptibility. Poultry Science, 77: 1130-1138.

Forbes, N.A., and Simpson, G.N. 1997. A review of viruses affecting raptors. Veterinary record. 141, 123-126.

Forrester, D.J., and Foster, G.W. 2008. Trichomonosis. In: Atkinson, C.T., Thomas, N.J. and Hunter, D.B. (Eds.) *Parasitic Diseases of Wild Birds*. Wiley-Blackwell. Pp: 120-153.

Gamino, V., and Höfle, U. 2013. Pathology and tissue tropism of natural West Nile virus infection in birds: a review. Veterinary Research, 44:39.

Garner, M.M.1989. Bumblefoot associated with poxvirus in a wild golden eagle (*Aquila chrysaetos*). Companion Animal Practice, 19: 17-20.

Greiner, E.C. 2008. Isospora, Atoxoplasma, and Sarcocystis. In: Atkinson, C.T., Thomas, N.J. and Hunter, D.B. (Eds.) *Parasitic Diseases of Wild Birds*. Wiley-Blackwell. Pp: 108-119.

Gómez-Muňoz, M., Martínez-Herrero, M., Sansano-Maestre, and Toledo, M. 2018. Oropharyngeal trichomonads in wild birds. In: Jenkins, O. (ed.). *Advances in Animal Science and Zoology*. 11:1-41.

Gosbell, M.C., Olaogun, O.M., Luk, K.H.Y., and Noormohammadi, A.H. 2020. Investigation of systemic isosporosis outbreaks in an aviary of greenfinch (*Carduelis chloris*) and goldfinch (*Carduelis carduelis*) and a possible link with local wild sparrows (*Passer domesticus*). Australian Veterinary Journal, 98(7): 338-344.

ICTV. 2018. International Committee on Taxonomy of Viruses. ICTV 2018 master species list (MSL33). Available from: <https://talk.ictvonline.org/files/master-species-lists/m/msl/7992> (Accessed March 2021)

Jansson, D.S. 2012. Aspergillosis. In Gavier-Widen, G., Duff, P.D. & Meredith, A. (Eds), *Infectious Diseases of Wild Mammals and Birds in Europe* (First edition pp. 455-461). Blackwell Publishing Ltd., UK

Kaleta, E.F., and Tadai, E.M.A. 2003. Avian host range of *Chlamydophila* spp. based on isolation, antigen detection and serology. Avian Pathology, 32(5): 435-462. Available from: doi: 10.1080/03079450310001593613.

Kearns, K.S. 2014. Avian aspergillosis. In: *IVIS Reviews in Veterinary Medicine*. I.V.I.S. (Ed.), International Veterinary Information Service, Ithaca, NY, [www.ivis.org](http://www.ivis.org)

Kocan, R.M. 1969. Various grains and liquid as potential vehicles of transmission for *Trichomonas gallinae*. Bulletin of the Wildlife Diseases Association, 5: 148-149.

Komar, N., Langevin, S., Hinten, S., Nemeth, N., Edwards, E., Hettler, D., Davis, B., Bowen, R., and Bunning, M. 2003. Experimental infection of North American birds with the New York 1999 strain of West Nile Virus. Emerging Infectious Diseases, 9(3): 311-322.

Kilpatrick, A.M., Daszak, P., Jones, M.J., Marra, P.P., and Kramer, L.D. 2006. Host heterogeneity dominates West Nile virus transmission. Proceedings of The Royal Society B, 273: 2327-2333. Doi:10.1098/rspb.2006.3575

Lafferty, K.D. et al. 2018. Local extinction of the Asian tiger mosquito (*Aedes albopictus*) following rat eradication on Palmyra Atoll. Biol. Lett. 14: 20170743. http://dx.doi.org/10.1098/rsbl.2017.0743

Lanciotti, R.S., Roehrig, J.T., Deubel, V., Smith, J., Parker, M., Steele, K., Crise, B., Volpe, K.E., Crabtree, M.B., Scherret, J.H., Hall, R.A., MacKenzie, J.S., Cropp, C.B., Panigrahy, B., Ostlund, E., Schmitt, B., Malkinson, M., Banet, C., Weissman, J., Komar, N., Savage, H.M., Stone, W., McNamara, T., and Gubler, D.J. 1999. Origin of the West Nile virus responsible for an outbreak of encephalitis in the north eastern United States. Science, 286:2333–2337.

Lanciotti, R.S., Ebel, G.D., Deubel, V., Kerst, A.J., Murri, S., Meyer, R., Bowen, M., McKinney, N., Morrill, W.E., Crabtree, M.B., Kramer, L.D., and Roehrig, J.T. 2002. Complete genome sequences and phylogenetic analysis of West Nile virus strains isolated from the United States, Europe, and the Middle East. Virology, 298: 96-105.

Locke, L.N. 1987. Aspergillosis. In: *Field Guide to Wildlife Diseases*. Friend, M. (Ed.). United States Department of the Interior Fish and Wildlife Service. Resource Publication 167: 145-150.

Mackenzie, J.S., and Williams, D.T. 2009. The zoonotic flaviviruses of Southern, South-Eastern and Eastern Asia, and Australasia: the potential for emergent viruses. Zoonoses and Public Health, 56: 338-356. doi: 10.1111/j.1863-2378.2008.01208.x

McMillan, M.C., and Petrak, M.L. 1989. Retrospective study of aspergillosis in pet birds. Journal of the Association of Avian Veterinarians, 3: 211-215.

Mohr, F., Betson, M., and Quintard, B. 2017. Investigation of the presence of *Atoxoplasma* spp. in blue crowned laughing thrush (*Dryonastes courtoisi*) adults and neonates. Journal of Zoo and Wildlife Medicine, 48: 1-6.

Munro, G. 2006. Outbreak of avian pox virus in gentoo penguins in the Falklands, February 2006. Sandy, UK: Falkland Conservation.

Oglesbee, B.L. 1997. Mycotic diseases. In: *Avian Medicine and Surgery*. 1st edn, Altman, R.B. (Ed.). Philadelphia, PA: W.B. Saunders Company, pp. 323-361.

Pal, M. 2017. *Chlamydophila psittaci* as an emerging zoonotic pathogen of global significance. International Journal of Vaccines and Vaccination, 4(3): 00080. Available from: doi: 10.15406/ijvv.2017.04.00080

Perrott, J.K., and Armstrong, D.P. 2011. *Aspergillus fumigatus* densities in relation to forest succession and edge effects: implications for wildlife health in modified environments. EcoHealth, 8: 290-300.

Phalen, D.N. 2000. Respiratory medicine of cage and aviary birds. Veterinary Clinics of North America: Exotic Animal Practice, 3: 423-452.

Reid, H.W., Weissenböck, H., and Erdélyi, K. 2012. Flavivirus Infections. In: *Infectious Diseases of Wild Mammals and Birds in Europe* (1st edition). Gavier-Widen, G., Duff, P.D., & Meredith, A. (Eds). Blackwell Publishing Ltd., UK. Pp: 128-145.

Riggs, G. 2012. Avian Mycobacterial Disease. In Miller, R.E., and Fowler, M. (Eds.) Fowler’s Zoo and Wild Animal Medicine 7. Pp. 266-274.

Ryley, J.F. 1973. Cytochemistry, physiology, and biochemistry. In: *The Coccidia. Eimeria, Isospora, Toxoplasma, and Related Genera* (Hammond, D.M. and Long, P.L., eds), pp. 145–181, University Park Press.

Savini, G., Capelli, G., Monaco, F., Polci, A., Russo, F., Di Gennaro, A., Marini, V., Teodori, L., Montarsi, F., Pinoni, C., Pisciella, M., Terregino, C., Marangon, S., Capua, I., and Lelli, R. 2012. Evidence of West Nile virus lineage 2 circulation in Northern Italy. Veterinary Microbioly, 158: 267–273.

Shearn-Bochsler, V., Green, D.E., Converse, K.A., et al. 2008. Cutaneous and diphtheritic avian poxvirus infection in a nestling southern giant petrel (*Macronectes giganteus*) from Antarctica. Polar Biology, 31: 569-573.

Smith, G.W., Ives, L.D., Nagelkerken, I.A. and Ritchie, K.B. 1996. Caribbean sea fan mortalities. Nature, 383: 487.

Speck, S. and Duff, J.P. 2012. Chlamydiacea infections. In: Gavier-Widen, G., Duff, P.D. & Meredith, A. (Eds), *Infectious Diseases of Wild Mammals and Birds in Europe* (First edition pp. 336-344). Blackwell Publishing Ltd., UK.

Sukon, P., Nam, N.H., Kittipreeya, P., Sara, A., Wawilai, P., Inchuai, R., and Weerakhun, S. 2021. Global prevalence of chlamydial infections in birds: A systematic review and meta-analysis. Preventive Veterinary Medicine, 192:105370.

Tasca, T., and De Carli, G. 2003. Scanning electron microscopy study of Trichomonas gallinae. Veterinary Parasitology, 118: 37-42.

Tell, L.A. 2005. Aspergillosis in mammals and birds: impacts on veterinary medicine. Medical Mycology Supplement, 1: S7-S73.

Travis, E.K., Junge, R.E., and Terrell, S.P. 2007. Infection with *Mycobacterium simiae* complex in four captive Micronesian kingfishers. Journal of the American Veterinary Medical Association, 230(10): 1524-1529.

Tripathy, D.N. 1993. Avipox viruses. In: McFerran, J.B. & McNulty, M.S. (eds.) *Virus infections of vertebrates – Virus infections of birds*. Elsevier Science Publishers B.V., Amsterdam, The Netherlands, pp. 5-15.

Tshai, S.S., Park, J.H., Hirai, K., and Itakura, C. 1992. Aspergillosis and candidiasis in psittacine and passeriform birds with particular reference to nasal lesions. Avian Pathology, 21: 699-709.

Valiakos, G., Touloudi, A., Iacovakis, C., Athanasiou, L., Birtsas, P., Spyrou, V., and Billinis, C. 2011. Molecular detection and phylogenetic analysis of West Nile virus lineage 2 in sedentary wild birds (Eurasian magpie), Greece, 2010. Euro Surveillance 2011, 16(18): 19862. May 5.

Vanderheyden, N. 1993. Aspergillosis in psittacine chicks. In*: Proceedings of the Annual Conference of the Association of Avian Veterinarians*. Jackson, G. (Ed.). Nashville, TN, USA. p: 207.

van Riper III, C., and Forrester, D.J. 2007. Avian pox. In: Thomas, N.J., Hunter, D.B. & Atkinson, C.T. (eds.) *Infectious Diseases of Wild Birds*. Blackwell Publishing, Iowa, pp. 131-176.

Wodak, E., Richter, S., Bago, Z., Revilla-Fernandez, S., Weissenboeck, H., Nowotny, N., and Winter, P. 2011. Detection and molecular analysis of West Nile virus infections in birds of prey in the eastern part of Austria in 2008 and 2009. Veterinary Microbioly, 149: 358–366.

Woodford, M.H., and Rossiter, P.B. 1994. Disease risks associated with wildlife translocations projects. In: *Creative Conservation: interactive management of wild and captive animals. Proceedings of the Sixth World Conference on Breeding Endangered Species*. Oleny, P.J.S., Mace, G. & Feistner, A.T.C. (Eds.). Chapman and Hall, London.

Xavier, M.O. 2008. A review of aspergillosis in penguins. Available online at <http://www.aspergillus.org.uk/secure/articles/aspergillo.pdf>

**Appendix S3.** Clinical examination including infectious hazard screening tests for young sihek.

| **Physical Examination at 20 days old** |
| --- |
| - Weight - Description of general appearance and attitude (bright, alert and responsive versus quiet, depressed and lethargic) - Examination of eyes, ears and nares - Examination of beak and oral mucous membrane - Examination of skin and feathers - Abdomen palpation - Auscultation heart/lungs/air sacs - Examination of cloaca and vent - Examination of wings for any skeletal deformities, range of motion and symmetry - Examination of feet and legs for any skeletal deformities and symmetry |
| **West Nile Virus screening** |
| - RT-PCR screening on DNA extracted from blood samples collected at 20 days old - RT-PCR screening on DNA extracted from cloacal swabs done at 20 days old |
| **Avian Poxvirus screening** |
| - RT-PCR screening on DNA extracted from blood samples collected at 20 days old |
| ***Aspergillus fumigatus* screening** |
| - No screening done |
| ***Chlamydophila psittaci* screening** |
| - PCR screening on DNA extracted from cloacal swab done at 20 days old |
| ***Trichomonas gallinae* screening** |
| - Visual inspection of open beak and mouth for lesions at 20 days old - Oral swab at 20 days old inoculated into a growth medium for flagellate protozoa. |
| ***Isospora* spp. screening** |
| - Three faecal samples collected from each sihek within a 5-day period starting at 18 days old with max of one per day. Samples then screened using a centrifugal faecal flotation test with Sheather’s sugar solution. - Examination of a Giemsa-stained blood smear from blood sampled at 20 days old. |
| ***Mycobacterium* Sp. screening** |
| - Source institutions not detected disease within their sihek for previous 12 months - RT-PCR screening of DNA extracted from three faecal samples collected as above. |

**Appendix S4.**

Summary of available prevalence, virulence and sample size data from published studies on each pathogen hazard.

**West Nile Virus**

| **Study** | **Species** | **Setting** | **Method** | **Prevalence** | **Mortality** |
| --- | --- | --- | --- | --- | --- |
| Ain-Najwa et al. (2020) | Various  (12 species) | Wild (Malaysia) | Sero  PCR | 29/155 (19%)  16/105 (15%) | N/A |
| Barbachano-Guerrero et al. (2019) | Various (21 species) | Wild (Mexico) | PCR | 16/200 (8%) | N/A |
| Bradley et al. (2008) | Various (27 species) | Wild (USA) | Sero | 73/499 (14.6%) | N/A |
| Caffrey et al. (2005) | American crow | Wild (USA) | Observation | N/A | ~72% overall |
| Cano-Terriza et al. (2015) | Various (4 species) | Zoo (Spain) | Sero | 3/142 (2.1%) | N/A |
| Carlson-Bremer et al. (2010) | American oystercatcher (*Haematopus palliatus palliatus*) | Wild (USA) | Sero | 0/34 (0%) | N/A |
| Clark et al. (2006) | Sage grouse | Lab (USA) | Experimental infection^1^ | 9/9 (100%) | 9/9 (100%) |
| Crosbie et al. (2008) | Various (7 corvid species) | Wild (USA) | PCR (dead birds) | 5240/8704 (60%) | N/A |
| Dusek et al. (2009) | Various (133 species) | Wild (USA) | Sero | 254/13403 (1.9%,  0-10% by species) sero+  19/13403 (0.1%) viremic | N/A |
| Dusek et al. (2010) | American kestrel (*Falco sparverius*), Burrowing owl (*Athene cunicularia*) | Wild (USA) | Sero | 173/208 (83%) kestrel;  36/116 (31%) owl | N/A |
| Dusek et al. (2012) | American kestrel (*Falco sparverius*) | Wild (USA) | Sero | 108/111 (97.3%) antibody+; 0/111 (0%) virus+ | N/A |
| Foss et al. (2015) | Various | Wild (USA) | PCR (dead) | 15864/47629 (33%, 0-56% by year)^2^ | N/A |
| Gancz et al. (2004) | Various (19 species) | Captive (Canada) | PCR + sero (carcasses in outbreak) | 79/85 (93%) of dead birds | 79/235 (34%) overall |
| Gibson et al. (2019) | Various (110 psittacine species) | Captive (Canada) | General post-mortem | 3/1850 (0.2%) | N/A |
| Hartup et al. (2008) | Various (8 Gruidae/crane species) | Captive (USA) | Sero | 18/110 (16%) | N/A |
| Hofmeister et al. (2016) | Various (4 duck species) | Wild (USA) | Sero | 148/1405 (10%) | N/A |
| Hubálek et al. (2008a) | Various^3^ (28 species) | Wild  (Czech Republic) | Sero | 13/391 (3.3%) | N/A |
| Hubálek et al. (2008b) | Various (11 species) | Wild (Poland) | Sero | 5/97 (5.2%) | N/A |
| Hull et al. (2006) | Various (3 hawk species) | Wild (USA) | Sero | 36/320 (11.3%) | N/A |
| Hull et al. (2010) | Various (3 raptor species) | Wild (USA) | Sero | 0/293 (0%) | N/A |
| Kilpatrick et al. (2013) | Carolina wren  Northern cardinal  Tufted titmouse | Wild (USA) | Sero | 17.5% wrens  34.8% cardinals  1.7% titmouse |  |
| Kilpatrick et al. (2013) | Carolina wren  Tufted titmouse | Lab (USA) | Experimental infection | N/A | 3/11 (27%) wrens  12/12 (100%) titmouse |
| Komar et al. (2003) | Various (25 species) | Lab | Experimental infection | 83/87 (96%) became viremic | 33-100% by species |
| Komar et al. (2005) | Various | Wild (USA) | Sero | 41/264 (15.5%) | N/A^4^ |
| Komar et al. (2013) | Various (17 species) | Wild (USA) | Sero | 144/300 (48%) | N/A |
| Kwan et al. (2010) | Various | Wild (USA) | PCR (dead birds);  sero (live birds) | 1972/5177 (38%) dead  1351/14107 (9.6%) live | N/A |
| Kwan et al. (2012) | Various (38 species) | Wild (USA) | Sero | 2267/22672 (10%) | N/A |
| Levine et al. (2013) | Various (41 passerine species) | Wild (USA) | Sero (viremia) | 6/630 (0.95%) |  |
| Loss et al. (2009) | Various (60 species) | Wild (USA) | Sero | 236/2061 (11.45, 3.5-20.5% by year) | N/A |
| Ludwig et al. (2002) | Various  (124 species) | Captive (USA) | Sero | 125/368 (34%) positive  27/125 (22%) infectious | 19/27 (22%) |
| Medeiros et al. (2014) | Various (13 species) | Wild (USA) | Sero | 120/1714 (7%) | N/A |
| McLean et al. (2001) | American crow | Lab (USA) | Experimental infection | 19/19 (100%) | 19/19 (100%) |
| Michel et al. (2019) | Various (143 species) | Wild (Germany) | Sero, PCR | 0/1709 (0%) | N/A |
| Morales-Betoulle et al. (2013) | Various (19 species) | Wild (Guatemala) | Sero | 121/985 (12.3%) | N/A |
| Naugle et al. (2005) | Sage grouse | Wild (USA) | PCR | N/A | 14%^5^ |
| Nemeth et al. (2006) | Various (4 raptors) | Lab (USA) | Experimental infection | 13/15 (87%) viremic | Euthanized |
| Nemeth et al. (2010) | Various (11 species) | Wild (Hawaii) | Sero | 0/1835 (0%) | N/A |
| Nemeth et al. (2016) | Various (153 species) | Captive + wild (USA) | General post-mortem | 6/827 (0.7%) | N/A |
| Nemeth et al. (2021) | Ruffed grouse (*Bonasa umbellus*) | Wild (USA) | Sero (dead birds) | 81/563 (14.4%, 2.8-22.6% by year) |  |
| O’Brien et al. (2010) | House sparrow (*Passer domesticus*) | Wild (USA) | PCR and/or plaque assays (nestlings);  Sero (juv/adults) | 7/173 (4%) nestlings;  26/189 (13.8%) juv/adults | N/A |
| Reisen et al. (2005); Fang and Reisen (2006) | American robin | Lab (USA) | Experimental infection | 2/5 (40%) low dose  7/7 (100%) high dose | 2/2 (100%) low dose  0/7 (0%) high dose |
| Reisen et al. (2009) | Various (ardeids) | Wild (USA) | PCR (dead birds) | 12/81 (15%) | N/A |
| Reisen et al. (2013) | Various (163 species) | Wild (USA) | PCR (dead birds) | 1092/3499 (31%, 2-51% by season) | N/A |
| Russell et al. (2014) | Bald eagle (*Haliaeetus leucocephalus*), golden eagle (*Aquila chryaetos*) | Wild (USA) | General post-mortem | 6/4407 (0.1%) | N/A |
| Straub et al. (2015) | Various (3 raptor species) | Wild (USA) | Sero | 116/272 (43%) | N/A |
| Straub et al. (2015) | California condor | Captive (USA) | Sero | 0/41 (0%) | N/A |
| Walker et al. (2007) | Sage grouse | Wild (USA) | Sero | 4.2% - 13.6%^6^ | 2.4% - 28.9%^5^ |
| Wheeler et al. (2009) | Various (>230 species) | Wild (USA) | Sero (live and dead birds) | 1695/25116 (6.7%) live  9040/27194 (33%) dead | N/A |
| Wheeler et al. (2009) | Various (184 birds 27 species) | Lab (USA) | Experimental infection | N/A | 0-100% by species |
| Wheeler et al. (2014) | American crow (*Corvus brachyrhynchos*) | Wild (USA) | PCR (dead birds) | 24/67 (35.8%) | N/A |
| Yaremych et al. (2004) | American crow | Wild (USA) | PCR | 5/156 (3.2%) | 19/28 (68%) |

^1^ vaccine trial: data refer to unvaccinated animals (5/6 vaccinated died)

^2^ Coraciiformes listed as comprising “2% or less of positive bird species” (belted kingfisher tested)

^3^ only study to include kingfisher (1/1 WNV-positive)

^4^ blue jay population reduced by estimated ~47%

^5^ modelled mortality for WNV-infected birds; cf. 4-8% for non-infected birds.

^6^ modelled mortality

**Avian poxvirus**

| **Study** | **Species** | **Setting** | **Method** | **Prevalence** | **Mortality** |
| --- | --- | --- | --- | --- | --- |
| Al Falluji et al. (1979) | Peacock | Zoo (Iraq) | Lesions (outbreak) | 45/60 (75%) | 8/45 (18%) |
| Aruch et al. (2007) | Various (10 species) | Wild (USA) | Lesions | 2/247 (0.8%) | N/A |
| Atkinson et al. (2010) | Various (9 species) | Wild (Hawaii) | Lesions | 164/1277 (12.8%) | N/A |
| Catroxo et al. (2009) | Various (3 passerine species) | Captive (Brazil) | Lesions (outbreak) | NA | 500/800 (62.5%) |
| Catroxo et al. (2012) | Bay-winged cowbird | Captive (Brazil) | Lesions (outbreak) | 45/60 (75%) | Euthanised |
| Donnelly and Crane (1984) | House sparrow | Captive (USA) | Lesions (outbreak) | 3/120 (2.5%) | Euthanised |
| Ellison et al. (2014) | Henslow’s sparrow | Wild (USA) | Lesions | 15/165 (9%) | N/A |
| Esteves et al. (2017) | Various (10 parrot species) | Captive (Brazil) | Lesions + PCR^1^  (outbreak) | 23/27 (85%) PCR+ w/ lesions  4/67 (5.9%) PCR+ w/o lesions | 3/27 (11%) |
| Fanke et al. (2011) | Eurasian cranes (*Grus grus*) | Wild (Germany) | Lesions | 6/167 (3.6%) | N/A |
| Gaudioso-Levita et al. (2015) | Various (11 species) | Wild (Hawaii) | Lesions | 13/785 (1.7%) | N/A |
| González-Hein et al. (2008) | Various (7 parrot species) | Captive (Chile) | Lesions | 50/188 (27%) | 11/50 (22%) |
| Hukkanen et al. (2003) | Gray-crowned rosy finch | Captive (USA) | Lesions (outbreak) | 12/16 (75%) | Euthanised |
| Johnson and Castro (1986) | Canary | Captive (USA) | Lesions (outbreak) | 165/200 (82.5%) | 145/165 (88%) |
| Kane et al. (2012) | Magellanic penguin | Wild (Argentina) | Lesions | 108/~54K (0.2%) | >18/108 (>17%) |
| Kulich et al. (2008) | Blackcap | Wild (Czech Republic) | Lesions + PCR^1^ | 9/244 (3.7%) | N/A |
| Lachish et al. (2012) | Various (6 passerine species) | Wild (UK) | Lesions + PCR/histo^1^ | 114/8048 (1.4%) | N/A |
| Le Loc'h et al. (2016) | Houbara bustard | Captive (UAE) | Lesions | 225/~16K (1.4%) | N/A |
| MacDonald et al. (2019) | Wild turkey (*Meleagris gallopavo*) | Wild (Canada) | Lesions + PCR^1^ (dead birds) | 5/215 (2.3%) | N/A |
| Moens et al. (2017) | Various (94 species) | Wild (Ecuador, French GY) | Lesions + PCR^1^ | 3/1830 (0.2%) | N/A |
| Palade et al. (2008) | Great tit | Wild (Hungary) | Lesions | 15/1819 (0.8%) | N/A |
| Ruiz-Martínez et al. (2016) | House sparrow | Wild (Spain) | Lesions | 85/2679 (3.2%)^2^ | N/A |
| Samuel et al. (2018) | Various (4 species) | Wild (Hawaii) | Active lesions, old lesions | 540/11743 (4.6%) active, 305/11743 (2.6%) old | N/A |
| Shivaprasad et al. (2009) | Canary | Captive (USA) | Lesions (outbreak) | >255/450 (>65%) | Euthanised |
| Smits et al. (2005) | Short-toed lark, Berthelot’s pitpit | Wild (Canary Islands) | Lesions (outbreak) | 534/1286 (41.5%) | N/A |
| Tarello (2008) | Various (4 *Falco* spp.) | Captive (Kuwait, Dubai) | Lesions (checks) | 92/3706 (2.5%) | 3/92 (3.3%) |
| Tikasingh et al. (1982) | Various (4 species) | Wild (Trinidad & Tobago) | Lesions | 174/9514 (1-7%)^3^ | N/A |
| van Riper III et al. (2002) | Various (11 species) | Wild (Hawaii) | Lesions | 421/3122 (13.5%) | N/A^4^ |
| Wingate et al. (1980) | White-tailed tropicbird | Wild (Bermuda) | Lesions | 2/590 (0.3%) | N/A |
| Work et al. (2015) | Nene (*Branta sandvicensis*) | Captive + wild (Hawaii) | Lesions (dead birds) | 1/300 (0.3%)^5^ | N/A |
| Wrobel et al. (2016) | Various (7 raptor species) | Wild (USA) | Lesions, sero | 2/142 (1.4%) lesions, 66/142 (46.5%) sero+ | N/A |
| Yanga et al. (2011) | Mourning dove (*Zenaida macroura*), Socorro ground dove (*Columbina passerina socorrensis*) | Wild (Mexico) | Lesions | 0/56 (0%) | N/A |
| Young and VanderWerf (2008) | Laysan albatross (*Phoebastria immutabilis*) | Wild (Hawaii) | Lesions (chicks only) | 83/162 (51%) | N/A |

^1^ Animals with or without lesions, then PCR

^2^ ~65% of 50 subsequently tested by PCR were confirmed AVP positive

^3^ Multi-year study: prevalence varied by year and season, including multiple outbreaks

^4^ 94 native birds (6.3% of those caught and 25.3% of those infected) had “heavy” infection

^5^ Secondary finding in emaciated bird

Also see global review by Williams et al. (2021).

***Chlamydophila psittaci***

| **Study** | **Species** | **Setting** | **Method** | **Prevalence** | **Mortality** |
| --- | --- | --- | --- | --- | --- |
| Astorga et al. (1994) | Various (13 waterfowl species) | Wild (Spain) | Sero | 95/712 (13.3%) | N/A |
| Carlos and Luyo (2018) | Various (macaws) | Captive (Peru) | Sero | 17/38 (44.7%) | N/A |
| Carlson-Bremer et al. (2010) | American oystercatcher (*Haematopus palliatus palliatus*) | Wild (USA) | Sero | 26/107 (24%) | N/A |
| Feng et al. (2016) | Various  (8 species) | Zoo (China) | PCR | 7/60 (11.7%) | N/A |
| de Freitas Raso et al. (2002) | Various (Amazon parrots) | Captive (Brazil) | Sero  DIF* | 34/44 (77.3%)  34/95 (35.8%) | N/A |
| de Freitas Raso et al. (2004) | Blue-fronted Amazon parrot (*Amazona aestiva*) | Wildlife rehab center (Brazil) | Observation + post-mortem | 58/58 (100%) (outbreak) | 56/58 (96.5%) |
| de Freitas Raso et al. (2012) | Various  (8 species) | Captive (Brazil) | PCR  Sero | 0/25 (0%)  4/25 (16%) | N/A |
| Gibson et al. (2019) | Various (110 psittacine species) | Captive (Canada) | General post-mortem | 29/1850 (1.6%) | N/A |
| Herrera et al. (2001) | Scarlet macaw | Captive (Costa Rica) | Sero | 16/128 (12.4%) | N/A |
| Kabeya et al. (2015) | Various  (131 sp.) | Zoo (Japan) | PCR | 48/668 (7.2%) | N/A |
| Križek et al. (2012) | Various | Captive (Croatia) | Sero | 70/411 (23.1%) overall  22/177 (12.4%) parrots | N/A |
| Maluping et al. (2007) | Various | Captive (Philippines) | Sero | 9/36 (25%) | N/A |
| Nemeth et al. (2016) | Various (153 species) | Captive + wild (USA) | General post-mortem | 15/827 (1.8%) | N/A |
| Ornelas-Eusebio et al. (2020) | Various (poultry species) | Captive (Mexico) | PCR | 104/879 (11.8%) | N/A |
| Padilla et al. (2004) | Galapagos dove  Rock pigeon | Wild (Galapagos Is.) | PCR | 6/102 (6%) dove  0/28 (0%) pigeon | N/A |
| Piasecki et al. (2012) | Various (34 parrot species) | Captive (Poland) | Sero | 16/156 (10.3%) | N/A |
| Straub et al. (2015) | Various (3 raptor species) | Wild (USA) | Sero | 8/159 (5%) | N/A |
| Straub et al. (2015) | California condor | Wild (USA) | Sero | 2/41 (4.88%) | N/A |
| Suksai et al. (2016) |  | Captive (Thailand) | PCR | 14/178 (7.9%) | N/A |
| Sukon et al. (2021) | Various  (global review) | Multiple | Multiple | 19.5% (16-23%) world  22% (12-36%) N. Am. | N/A |
| Yanga et al. (2011) | Mourning dove (*Zenaida macroura*), Socorro ground dove (*Columbina passerina socorrensis*) | Wild (Mexico) | PCR | 0/56 (0%) | N/A |

* DIF: direct immunofluorescence

***Trichomonas gallinae***

| **Study** | **Species** | **Setting** | **Method** | **Prevalence** | **Mortality** |
| --- | --- | --- | --- | --- | --- |
| Arfin et al. (2019) | Domestic pigeon | Captive (Bangladesh) | Wet mount | 27/47 (60%) | N/A |
| Baker (1996) | Budgerigar | Captive (UK) | Histology | 121/933 (12.9%) dead  16/469 (3.4%) euthanased | N/A |
| Borji et al. (2011) | Domestic pigeon | Captive + wild (Iran) | Culture | 156/418 (37%) | N/A |
| Brobey et al. (2017) | Various (24 species) |  | PCR | 11/116 (9%) | N/A |
| Bunbury et al. (2007) | Various (3 columbid species) | Wild (Mauritius) | Lesions + culture | 131/296 (44%) | N/A |
| Bunbury et al. (2008) | Pink Pigeon | Wild (Mauritius) | Lesions + culture | 213/426 (50%) | N/A^1^ |
| Dudek et al. (2018) | Golden Eagle | Wild (N America) | PCR | 25/138 (18%) | N/A |
| Gibson et al. (2019) | Various (110 psittacine species) | Captive (Canada) | General post-mortem | 1/1850 (0.1%) | N/A |
| Höfle et al. (2004) | Common wood pigeon | Wild (Spain) | PCR on carcasses (outbreak) | N/A | ~2600/17000 overall |
| Krone et al. (2005) | Northern goshawk | Wild (Germany) | Lesions | 175/269 (65%) | 2/175 (1.1%) |
| Marx et al. (2017) | Various (4 columbid species) | Wild (southern Europe) | Culture + PCR | 208/281 (74%) | N/A |
| McKeon et al. (1997) | Various (5 species) | Captive (Australia) | Culture | 70/525 (13%) | N/A |
| Niedringhaus et al. (2019) | Barn owl (*Tyto alba*), Barred owl (*Strix varia*) | Wild (USA) | Lesions + culture (dead birds) | 2/26 (8%) barn,  4/22 (18%) barred | N/A |
| Padilla et al. (2004) | Galapagos dove  Rock pigeon | Wild (Galapagos Is.) | PCR | 0/102 (0%) dove  8/18 (44%) pigeon | N/A |
| Quillfeldt et al. (2018) | Various (35 species) | Wild (Germany) | PCR | 157/440 (35.6%) | N/A |
| Rosenfield et al. (2002) | Cooper’s Hawk | Wild (USA) | Culture | 3/110 (2.7%) | 0/3 (0%) |
| Sansano-Maestre et al. (2009) | Domestic pigeon  Raptors (15 species) | Captive (Spain) | PCR | 274/612 (45%) pigeons  20/102 (19.6%) raptors | 0 pigeons  4/20 (20%) raptors |
| Santos et al. (2019) | Rock doves  Wild columb.  Bonelli eagle | Captive + wild (Spain) | PCR | 175/256 (68%) rock  23/38 (61%) wild  1/16 (6.3%) eagles | N/A |
| Schulz et al. (2005) | Mourning dove | Wild (USA) | Culture | 226/4052 (5.6%) | N/A |
| Stockdale et al. (2015) | European turtle dove | Wild (UK) | Culture + PCR | 25/25 (100%) | >5/25 (25%) |
| Villanúa et al. (2006) | Common wood pigeon | Wild (Spain) | Lesions + culture | 31/91 (34%) | N/A |
| Yanga et al. (2011) | Mourning dove (*Zenaida macroura*), Socorro ground dove (*Columbina passerina socorrensis*) | Wild (Mexico) | Lesions | 0/56 (0%) | N/A |
| Zu Ermgassen et al. (2016) | Various (18 families) | Wild (UK – surveillance) | PCR | 6/275 (2.2%) | N/A |

^1^ significantly lower survival for birds which tested negative in >3 consecutive occasions (89.7% vs 66.7%)

**Mycobacterium**

| **Study** | **Species** | **Setting** | **Method** | **Prevalence** | **Mortality** |
| --- | --- | --- | --- | --- | --- |
| Abda et al. (2015) | Domestic chicken (*Gallus domesticus*) | Captive (Ethiopia) | Intradermal tuberculin test +_lesions + culture + stain + PCR | 11/260 (4.23%) | N/A |
| Algammal et al. (2021) | Various (domestic) | Captive (Egypt) | PCR | 10/170 (5.9%) | N/A |
| Borovská et al. (2011) | Various (14 species of Passeriformes, Falconiformes) | Wild (Slovakia) | PCR | 50/650 (7.7%) | N/A |
| Corn et al. (2005) | Common snipe, House sparrow, European starling | Wild (USA) | Culture | 9/101 (8.9%) (M. a. paratuberculosis) | N/A |
| Daoust et al. (2021) | Various (4 seabird species) | Wild (USA) | Culture + stain | 1/244 (0.4%) | N/A |
| De La Cruz Baltzar (2021) | Various (14 species) | Captive (Mexico) | Staining | 1/65 (4.6%) | N/A |
| Ebani et al. (2021) | Various (15 species) |  | Fecal sample PCR | 0/121 (0%) | N/A |
| Fanke et al. (2011) | Eurasian cranes (*Grus grus*) | Wild (Germany) | Lesions | 2/167 (1.2%) | N/A |
| Gaukler et al. (2009) | European starling (*Sturnus vulgaris*) | Wild (USA) | Culture + staining + PCR | 0/64 (0%) fecal M. a. paratuberculosis; 15/64 (23%) acid-fast+ | N/A |
| Gibson et al. (2019) | Various (110 psittacine species) | Captive (Canada) | General post-mortem | 45/1850 (2.4%) | N/A |
| Hodge et al. (2019) | Brolga (*Antigone rubicunda*) | Captive (Australia) | Culture | 5/7 (71%) | N/A |
| Millán et al. (2010) | Various raptors (12 species) | Wildlife rehab center (Spain) | Lesions + PCR | 14/589 (2.4%) | N/A |
| Moravkova et al. (2011) | Various | Captive + wild (Czech Republic) | Culture + PCR | 45/259 (17%)^2^ | N/A |
| Nemeth et al. (2016) | Various (153 species) | Captive + wild (USA) | General post-mortem | 5/827 (0.6%) | N/A |
| Nugent et al. (2011) | Various (6 species) | Wild (NZ) | Culture | 4/32 (12.5%) | N/A |
| Palmieri et al. (2013) | Various (Psittaciformes spp.) | Captive (USA) | Culture + stain + PCR (dead birds) | 123/9241 (1.33%) | N/A |
| Rhim et al. (2018) | Spot-billed duck (*Anas poecilorhyncha*), Mallard (*Anas platyrhynchos*) | Wild (Korea) | PCR | 44/128 (34.4%) | N/A |
| Sattar et al. (2021) | Various (mostly *Gallus domesticus* chicken) | Captive + wild/free-ranging domestic (Malaysia) | Culture, PCR | 6/296 (2%) culture, 6/242 (2.5%) PCR | N/A |
| Schmitz et al. (2018) | Various (7 Passeriformes, Psittaciformes species) | Captive (Germany) | PCR | 71/170 (42%) overall (0-91% by aviary) | N/A |
| Witte et al. (2008) | Various (894 species) | Captive (USA) | Culture + staining | 172/13976 (1.2%)^1^ | N/A |

^1^ Coraciiformes: 11/812 (1.4%)

^2^ Only species to test positive was common pheasant (45/226 = 19.9%; all captive)

***Aspergillus fumigatus***

| **Study** | **Species** | **Setting** | **Method** | **Prevalence** | **Mortality** |
| --- | --- | --- | --- | --- | --- |
| Alley et al. (1999) | Hihi (*Notiomystis cincta*) | Wild (NZ) | Observation (carcass not recovered) + lesions (dead birds) | >14/31 (>45%) dead birds (8/31 = 26% by observation, 6/31 = 19% by lesions) | N/A |
| Astorga et al. (1994) | Various (13 waterfowl species) | Wild (Spain) | Sero | 8/712 (1.1%) | N/A |
| Atasever and Gümüşsoy (2004) | European starling (*Sturnus vulgaris*) | Captive (Turkey) | N/A | Experimental infection | 18/18 (100%) |
| Beer et al. (1963) | Pink-footed goose (Anser brachyrhynchus Baillon), Canada goose (Branta c. canadensis L.), Herring gull (Larus a. argentatus L.) | Wild (UK) | Culture (live birds) | 86/1188 (7.2%) pink-footed goose,  4/61 (6.6%) Canada goose, 13/102 (12.7%) herring gull | N/A |
| Beernaert et al. (2008) | Racing pigeon (*Columba livia domestica*) | Captive (Germany) | N/A | Experimental infection | 14/20 (70%)^1^ |
| Brand et al. (1988) | Various (30 species) | Wild (USA) | Lesions + culture (dead and moribund birds) | >63/257 (>24.5%) | N/A |
| Brown et al. (1992) | Various (3 swan species) | Wild (UK) | General post-mortem | 17/356 (4.8%) | N/A |
| Carlson-Bremer et al. (2010) | American oystercatcher (*Haematopus palliatus palliatus*) | Wild (USA) | Sero (antibodies + antigens) | 38/95 (40%) antibodies, 28/88 (32%) antigens | N/A |
| Chaudhary and Sadana (1988) | Japanese quail (*Coturnix japonica*) | Captive (¿) | N/A | Experimental infection (chicks) | 20% |
| Chege et al. (2013) | Cape vulture (*Gyps coprotheres*) | Zoo (South Africa) | Lesions + culture (outbreak, live and dead birds) | 5/6 (83%) | 3/5 (60%)^2^ |
| Cheng et al. (2020) | White leghorn chicken | Captive (China) | N/A | Experimental infection (chicks) | 11/25 (44%) |
| Cork et al. (1999) | Hihi (*Notiomystis cincta*) | Captive + wild (NZ) | Lesions + culture^3^ (dead birds) | >11/38 (29%) | N/A |
| Da Silva Filho et al. (2015) | Magellanic penguin (*Spheniscus magellanicus*) | Wildlife rehab center (Brazil) | Lesions + culture (dead birds) | 66/327 (20%) prevalence, 48.5% proportionate mortality^4^ | N/A |
| Fanke et al. (2011) | Eurasian cranes (*Grus grus*) | Wild (Germany) | Lesions + culture (dead birds) | 7/167 (4.2%) | N/A |
| Gartrell et al. (2014) | New Zealand dotterel (*Chadrius obscurus aquilonius*) | Wildlife rehab center (NZ) | Observation + culture | 7/60 (12%) | 6/7 (86%)^5^ |
| German et al. (2002) | Various (3 penguin species) | Zoo (UK) | Sero | 57/61 (93%)^6^ | N/A |
| Gibson et al. (2019) | Various (110 psittacine species) | Captive (Canada) | General post-mortem | 32/1850 (1.7%) | N/A |
| Goetting et al. (2013) | Japanese quail (*Coturnix japonica*) | Captive (USA) | N/A | Experimental infection | 41/90 (46%) overall (0-88% by dosage) |
| Graczyk and Cockrem (1995) | Various (4 penguin species) | Wild (NZ, Antarctica) | Sero^3^ | 110/184 (60%) | N/A |
| Hereba et al. (2016) | Ostrich | Captive (Saudi Arabia) | N/A | Experimental infection | 3/10 (30%) overall (0-60% by treatment group) |
| Lagerquist et al. (1994) | Trumpeter swan (*Cygnus buccinator*), Tundra swan (*Cygnus columbianus*) | Wild (USA) | Lesions (dead birds) | 23/136 (17%) | N/A |
| Melo et al. (2020) | Black-browed albatross (*Thalassarche melanophris*), Atlantic yellow-nosed albatross (*Thalassarche chlororhynchos*) | Wildlife rehab center (Brazil) | Lesion + culture (dead bird) | 3/32 (9.4%) prevalence, 3/14 (21%) proportionate mortality^4^ | N/A |
| Nakeeb et al. (1981) | Peruvian penguin (*Spheniscus humboldti*) | Zoo (USA) | Observation + culture (outbreak) | 4/4 (100%) | 3/4 (75%)^7^ |
| Naldo and Samour (2004) | Various (6 *Falco* spp.) | Captive (Saudia Arabia) | Lesions + culture (live and dead birds) | 152/6838 (2.2%) | 13/152 (8.6%) |
| Nemeth et al. (2016) | Various (153 species) | Captive + wild (USA) | General post-mortem | 27/827 (3.3%) | N/A |
| Olias et al. (2010) | White stork (*Ciconia ciconia*) | Wild (Germany) | Lesions + culture + PCR (dead chicks) | >22/101 (>22%)^8^ | N/A |
| Olsen et al. (1997) | Whooping crane (*Grus americana*) | Captive (USA) | General post-mortem | 7/103 (6.8%) | N/A |
| Redig et al. (1980) | Goshawk (*Accipiter gentilis atricapillus*) | Wild (USA) | Culture (live birds) | 30/105 (29%) | N/A |
| Russell et al. (2014) | Bald eagle (*Haliaeetus leucocephalus*), golden eagle (*Aquila chryaetos*) | Wild (USA) | General post-mortem | 50/4407 (1.1%) | N/A |
| Sidor et al. (2003) | Common loon (*Gavia immer*) | Wild (USA) | Lesions (dead birds) | 14/522 (2.7%) confirmed, 37/522 (7.1%) suspected | N/A |
| Souza et al. (2005) | Trumpeter swan (*Cygnus buccinator*), Tundra swan (*Cygnus columbianus*) | Wild (USA) | Lesions (dead birds) | 64/400 (16%) | N/A |
| Van Waeyenberghe et al. (2012) | Racing pigeon (*Columba livia domestica*), Gyr-Saker hybrid falcon (*Falco rusticolus x F. cherrug*) | Captive (Belgium) | N/A | Experimental infection | 2/15 (13%) pigeon overall (0-40% by dosage), 0/15 (0%) falcon |
| Work et al. (2015) | Nene (*Branta sandvicensis*) | Captive + wild (Hawaii) | Lesions (dead birds) | 6/300 (2%) | N/A |
| Xavier et al. (2007) | Magellanic penguin (*Spheniscus magellanicus*) | Wildlife rehab center (Brazil) | Lesions + culture (dead birds) | 5/52 (9.6%), 42% proportionate mortality^4^ | N/A |

^1^ Proportion of observed deaths attributed to aspergillosis^2^ All individuals treated with enrofloxacin, itraconazole, terbinafine hydrochloride, and silymarin

^3^ Non-specific *Aspergillus spp.*

^4^ Total sample included 7 chicks with confirmed but unidentified fungal infection

^5^ 2 of 4 birds treated with amphotericin B (1 survived, 1 died)

^6^ “Detection of immunoglobin did not correlate with clinical disease”

^7^ Half of all birds given dexamethasone in addition to A. fumigatus

^8^ Only one bird responded to treatment; diagnosis based on clinical signs (not confirmed)

***Isospora* spp.**

| **Study** | **Species** | **Setting** | **Method** | **Prevalence** | **Mortality** |
| --- | --- | --- | --- | --- | --- |
| Atkinson et al. (2016) | Various (16 species) | Wild (American Samoa) | Blood smear (Atoxoplasma) | 7/766 (0.9%)^1^ | N/A |
| Borges et al. (2019) | Double-collared seedeater (*Sporophila frontalis*), Uniform finch (*Haplospiza unicolor*) | Wild (Brazil) | Fecal flotation | 5/17 (30%) | N/A |
| Cunha et al. (2008) | Various (21 Cracidae species) | Captive (Brazil) | Fecal microscopy | 0/84 (0%) | N/A |
| Dadam et al. (2019) | House sparrow (*Passer domesticus*) | Wild (UK) | Culture | 75/242 (31%) | N/A |
| Delgado-Velez et (2015) | Superb fairy-wren (*Malarus cyaneus*), Red-browed finch (*Neochima temporalis*) | Wild (Australia) | Fecal microscopy | 121/278 (44%) | N/A |
| Gibson et al. (2010) | House sparrow (*Passer domesticus*) | Wild (USA) | Fecal microscopy | 158/201 (79%) | N/A |
| Globokar et al. (2017) | Various | Captive (Germany) | Fecal flotation | 149/10356 (1%) | N/A |
| Golemansky (2011) | Various (3 penguin species) | Wild (Antarctica) | Fecal flotation | 54/360 (15%) | N/A |
| Ibañez et al. (2016) | European starling (*Sturnus vulgaris*) nestlings | Wild (Argentina) | Fecal microscopy | 18/71 (25.35%) | N/A |
| Keeler et al (2011) | Rufous-and-white wren (*Thryothorus rufalbus*), Plain wren (*Cantorchilus modestus*) | Wild (Costa Rica) | Fecal microscopy | 19/21 (91%) rufous-and-white wren,  5/7 (71%) plain wren | N/A |
| Keeler et al. (2012) | Various (7 Turdidae species) | Wild (Costa Rica) | Fecal microscopy | 21/84 (25%) | N/A |
| Keeler et al. (2014) | Rufous-capped warbler (*Basileuterus rufifrons*), Ovenbird (*Seiurus aurocapilla*) | Wild (Costa Rica) | Fecal microscopy | 7/21 (33%) | N/A |
| Ledwoń et al. (2008) | Budgerigars (*Melopsittacus undulatus*) | Captive (Poland?) | N/A | Experimental infection | 48 |
| Lugarini et al. (2018) | Various | Wild (Brazil) | Fecal flotation | 1/48 (2%) | N/A |
| Oyarzún-Ruiz et al. (2021) | House sparrow (*Passer domesticus*) | Wild (Chile) | Fecal microscopy | 0/108 (0%) | N/A |
| Pérez Cordón et al. (2009) | Various | Zoo (Spain) | Fecal microscopy | 20/984 (2%) | N/A |
| Schoener et al. (2013) | Various (6 species) | Wild (New Zealand) | Fecal flotation, PCR (coccidia) | 85/316 (27%) flotation, 16/65 (25%) PCR | N/A |
| Titilincu et al. (2009) | Peacock (*Pavo cristatus*) | Captive (Romania) | Fecal microscopy | 3/90 (3.3%) | N/A |
| Tung et al. (2007) | Various (6 species) | Captive | Experimental infection | 4//32 (12.5%)^2^ | 4/4 (100%)^2^ |
| Vianna Cardozo et al. (2019) | Various | Wildlife rehab center (Portugal) | Fecal flotation | 1/89 (1.1%) | N/A |

^1^ Collared kingfisher: 0/74 (0%)

^2^ 6 species inoculated but only one (Russet sparrow, *Passer rutilans*) showed clinical or histopathological signs of infection (with 100% mortality); authors suggest species-specificity of I. michaelbakeri

**References listed**

Abda S, Mamo G, Worku A, Ameni G. 2015. Preliminary Study on Avian Tuberculosis and Associated Risks in Domestic Chickens at Shashemene District, Ethiopia. Journal of Biology and Medical Sciences **3**:13–23.

Ain-Najwa MY, Yasmin AR, Omar AR, Arshad SS, Abu J, Mohammed HO, Kumar K, Loong SK, Rovie-Ryan JJ, Mohd-Kharip-Shah A-K. 2020. Evidence of West Nile virus infection in migratory and resident wild birds in west coast of peninsular Malaysia. One Health **10**:100134.

Al Falluji M, Tantawi H, ALBANA A, Al Sheikhly S. 1979. Pox infection among captive peacocks. Journal of wildlife diseases **15**:597-600.

Algammal AM, Hashem HR, Al-otaibi AS, Alfifi KJ, El-dawody EM, Mahrous E, Hetta HF, El-Kholy AW, Ramadan H, El-Tarabili RM. 2021. Emerging MDR-Mycobacterium avium subsp. avium in house-reared domestic birds as the first report in Egypt. BMC Microbiology **21**:1–11.

Alley M, Castro I, Hunter J. 1999. Aspergillosis in hihi (Notiomystis cincta) on Mokoia Island. New Zealand Veterinary Journal **47**:88–91.

Andres Delgado-Velez C. 2015. Spatial patterns of bird-parasite interactions along an urbanisation gradient. University of Wollongong, Wollongong, Australia.

Arfin S, Sayeed MA, Sultana S, Dash AK, Hossen ML. 2019. Prevalence of *Trichomonas gallinae* infection in Pigeon of Jessore District, Bangladesh. Journal of Advanced Veterinary and Animal Research **6**:549.

Aruch S, Atkinson CT, Savage AF, LaPointe DA. 2007. Prevalence and distribution of pox-like lesions, avian malaria, and mosquito vectors in Kīpahulu valley, Haleakalā National Park, Hawai’i, USA. Journal of Wildlife Diseases **43**:567–575.

Atasever A, Gümüşsoy KS. 2004. Pathological, Clinical and Mycological Findings in Experimental Aspergillosis Infections of Starlings. Journal of Veterinary Medicine Series A **51**:19–22.

Atkinson C, Utzurrum R, Seamon J, Schmaedick M, LaPointe D, Apelgren C, Egan A, Watcher-Weatherwax W. 2016. Effects of climate and land use on diversity, prevalence, and seasonal transmission of avian hematozoa in American Samoa. Hilo, USA. Available from http://dspace.lib.hawaii.edu/handle/10790/2624 (accessed August 30, 2021).

Baker J. 1996. Causes of mortality and morbidity in exhibition budgerigars in the United Kingdom. Veterinary record **139**:156-162.

Barbachano-Guerrero A, Vásquez-Aguilar AA, Aguirre AA, Norzagaray AAZ-, Gonzalez EC, Terrazas AL, Faisal JLA-. 2019. West Nile Virus prevalence in wild birds from Mexico. Journal of Wildlife Diseases **55**:425–431.

Beer J V. 1963. The incidence of aspergillus fumigatus in the throats of wild geese and gulls. Medical Mycology **2**:238–247.

Beernaert LA, Pasmans F, Haesebrouck F, Martel A. 2008. Modelling Aspergillus fumigatus infections in racing pigeons (Columba livia domestica). Avian Pathology **37**:545–549.

Borji H, Razmi G, Movassaghi A, Moghaddas E, Azad M. 2011. Prevalence and pathological lesion of *Trichomonas gallinae* in pigeons of Iran. Journal of Parasitic Diseases **35**:186-189.

Borovská P, Kabát P, Ficová M, Trnka A, Svetlíková D, Betáková T. 2011. Prevalence of avian influenza viruses, Mycobacterium avium, and Mycobacterium avium, subsp. paratuberculosis in marsh-dwelling passerines in Slovakia, 2008. Biologia **66**:282–287.

Bradley CA, Gibbs SEJ, Altizer S. 2008. Urban land use predicts West Nile Virus exposure in songbirds. Ecological Applications **18**:1083–1092.

Brand CJ, Windingstad RM, Siegfried LM, Duncan RM, Cook RM. 1988. Avian Morbidity and Mortality from Botulism, Aspergillosis, and Salmonellosis at Jamaica Bay Wildlife Refuge, New York, USA. Colonial Waterbirds **11**:284.

Brobey B, Kucknoor A, Armacost J. 2017. Prevalence of Trichomonas, Salmonella, and Listeria in wild birds from Southeast Texas. Avian Diseases **61**:347–352.

Brown M, Brown MJ, Linton E, Rees EC. 1992. Causes of mortality among wild swans in Britain. Wildfowl **43**:70–79.

Bunbury N, Jones C, Greenwood A, Bell D. 2007. *Trichomonas gallinae* in Mauritian columbids: implications for an endangered endemic. Journal of Wildlife Diseases **43**:399-407.

Bunbury N, Jones C, Greenwood A, Bell D. 2008. Epidemiology and conservation implications of *Trichomonas gallinae* infection in the endangered Mauritian pink pigeon. Biological Conservation **141**:153-161.

Caffrey C, Smith SC, Weston TJ. 2005. West Nile virus devastates an American crow population. The Condor **107**:128-132.

Cano-Terriza D, Guerra R, Lecollinet S, Cerdà-Cuéllar M, Cabezón O, Almería S, García-Bocanegra I. 2015. Epidemiological survey of zoonotic pathogens in feral pigeons (*Columba livia* var. *domestica*) and sympatric zoo species in Southern Spain. Comparative immunology, microbiology and infectious diseases **43**:22-27.

Carlos N, Luyo EP. 2018. Seroprevalence of *Chlamydia psittaci* in captive macaws (*Ara* spp.) in the department of Lima, Peru. Ciência Animal Brasileira **19**.

Carlson-Bremer D et al. 2010. Health assessment of American oystercatchers (Haematopus palliatus palliatus) in Georgia and South Carolina. Journal of Wildlife Diseases **46**:772–780.

Catroxo M, Martins A, Petrella S, Milanelo L 2012. Detection of Poxvirus Using Transmission Electron Microscopy Techniques During Outbreak in Bay-Winged Cowbird (Gnorimopsar Chopi). IntechOpen.

Catroxo M, Pongiluppi T, Melo N, Milanelo L, Petrella S, Martins A, Rebouças M. 2009. Identification of poxvirus under transmission electron microscopy during outbreak period in wild birds, in São Paulo, Brazil. International Journal of Morphology **27**.

Chaudhary S, Sadana J. 1988. Experimental aspergillosis in Japanese quails (Coturnix coturnix japonica). Clinical signs and haematological changes. Mycopathologia **102**:179–184.

Chege S, Howlett J, Qassimi M Al, Toosy A, Kinne J, Obanda V. 2013. Opportunistic infection of Aspergillus and bacteria in captive Cape vultures (Gyps coprotheres). Asian Pacific Journal of Tropical Biomedicine **3**:401–406.

Cheng Z, Li M, Wang Y, Chai T, Cai Y, Li N. 2020. Pathogenicity and Immune Responses of Aspergillus fumigatus Infection in Chickens. Frontiers in Veterinary Science **0**:143.

Clark L, Hall J, McLean R, Dunbar M, Klenk K, Bowen R, Smeraski CA. 2006. Susceptibility of greater sage-grouse to experimental infection with West Nile virus. Journal of Wildlife Diseases **42**:14-22.

Cork SC, Alley MR, Johnstone AC, Stockdale PHG. 1999. Aspergillosis and other causes of mortality in the stitchbird in New Zealand. Journal of Wildlife Diseases **35**:481–486.

Corn JL, Manning EJB, Sreevatsan S, Fischer JR. 2005. Isolation of Mycobacterium avium subsp. paratuberculosis from free-ranging birds and mammals on livestock premises. Applied and Environmental Microbiology **71**:6963–6967.

Crosbie SP et al. 2008. Early Impact of West Nile Virus on the Yellow-Billed Magpie (Pica Nuttalli). The Auk **125**:542–550

Cunha ALB, Mendonça FS, Oliveira RA, Baratella-Evêncio L, Oliveira-Filho RM, Simões RS, Simões MJ, Evêncio-Neto J. 2008. Prevalence of Endoparasites in Faecal Samples of Cracids Bred in Captivity at the Parque Dois Irmãos, Recife, Pernambuco, Brazil. Acta Veterinaria Brno **77**:387–392.

Dadam D, Robinson RA, Clements A, Peach WJ, Bennett M, Rowcliffe JM, Cunningham AA. 2019. Avian malaria-mediated population decline of a widespread iconic bird species. Royal Society Open Science **6**. The Royal Society.

Daoust P-Y, Wong S, Holland E, Lucas ZN. 2021. Pathology of Northern fulmars (Fulmarus glacialis) and shearwaters beached on Sable Island, Nova Scotia, Canada. Journal of Wildlife Diseases **57**:601–611.

Da Silva Filho RP, Xavier MO, Martins AM, Ruoppolo V, Mendoza-Sassi RA, Adornes AC, Cabana ÂL, Meireles MCA, Cabana ÂL. M. 2015. Incidence density, proportionate mortality, and risk factors of Aspergillosis in Magellanic penguins in a rehabilitation center from Brazil. Journal of Zoo and Wildlife Medicine **46**:667–674.

de Freitas Raso T, Ferreira VL, Teixeira RHF, Pinto AA. 2012. Survey on *Chlamydophila psittaci* in captive ramphastids in São Paulo State, Brazil. SciELO Brasil.

de Freitas Raso T, Júnior ÂB, Pinto AA. 2002. Evidence of *Chlamydophila psittaci* infection in captive Amazon parrots in Brazil. Journal of Zoo and Wildlife Medicine **33**:118-121.

de Freitas Raso T, Godoy SN, Milanelo L, Souza CAI de, Matuschima ER, Araújo JP, Pinto AA. 2004. An outbreak of Chlamydiosis in captive blue-fronted Amazon parrots (Amazona aestiva) in Brazil. Journal of Zoo and Wildlife Medicine **35**:94–96.

De La Cruz Baltazar E. 2021. Presence of Mycobacterium sp in the feces of captive wild birds in three municipalities of the State of Jalisco. Journal of the Selva Andina Animal Science **8**:22–29.

Donnelly T, Crane L. 1984. An epornitic of avian pox in a research aviary. Avian diseases:517-525.

Dudek BM, Kochert MN, Barnes JG, Bloom PH, Papp JM, Gerhold RW, Purple KE, Jacobson KV, Preston CR, Vennum CR. 2018. Prevalence and risk factors of *Trichomonas gallinae* and trichomonosis in Golden eagle (*Aquila chrysaetos*) nestlings in Western North America. Journal of wildlife diseases **54**:755-764.

Dusek RJ, McLean RG, Kramer LD, Ubico SR, Dupuis II AP, Ebel GD, Guptill SC. 2009. Prevalence of West Nile virus in migratory birds during spring and fall migration.

Dusek RJ, Iko WM, Hofmeister EK. 2010. Occurrence of West Nile Virus infection in raptors at the Salton Sea, California. Journal of Wildlife Diseases **46**:889–897.

Dusek RJ, Iko WM, Hofmeister EK. 2012. Prevalence and effects of West Nile Virus on wild American Kestrel (Falco sparverius) populations in Colorado. Pages 45–54 in E. Paul, editor. Emerging Avian Disease. University of California Press, Berkeley, USA.

Ebani VV, Guardone L, Bertelloni F, Perrucci S, Poli A, Mancianti F. 2021. Survey on the Presence of Bacterial and Parasitic Zoonotic Agents in the Feces of Wild Birds. Veterinary Sciences **8**:171.

Ellison KS, Hofmeister EK, Ribic CA, Sample DW. 2014. Relatively high prevalence of pox-like lesions in Henslow's sparrow (*Ammodrammus henslowii*) among nine species of migratory grassland passerines in Wisconsin, USA. Journal of wildlife diseases **50**:810-816.

Esteves FC, Marín SY, Resende M, Silva AS, Coelho HL, Barbosa MB, D’Aparecida NS, de Resende JS, Torres AC, Martins NR. 2017. Avian pox in native captive Psittacines, Brazil, 2015. Emerging infectious diseases **23**:154.

Fang Y, Reisen WK. 2006. Previous infection with West Nile or St. Louis encephalitis viruses provides cross protection during reinfection in house finches. The American journal of tropical medicine and hygiene **75**:480-485.

Fanke J, Wibbelt G, Krone O. 2011. Mortality factors and diseases in free-ranging Eurasian cranes (Grus grus) in Germany. Journal of Wildlife Diseases **47**:627–637.

Feng Y, Feng Y-m, Zhang Z-h, Wu S-x, Zhong D-b, Liu C-j. 2016. Prevalence and genotype of *Chlamydia psittaci* in faecal samples of birds from zoos and pet markets in Kunming, Yunnan, China. Journal of Zhejiang University-SCIENCE B **17**:311-316.

Foss L, Padgett K, Reisen WK, Kjemtrup A, Ogawa J, Kramer V. 2015. West Nile Virus-related trends in avian mortality in California, USA, 2003-12. Journal of Wildlife Diseases **51**:576–588.

Gancz AY, Barker IK, Lindsay R, Dibernardo A, McKeever K, Hunter B. 2004. West Nile virus outbreak in north American owls, Ontario, 2002. Emerging infectious diseases **10**:2135.

Gartrell BD et al. 2013. Captive husbandry and veterinary care of northern New Zealand dotterels (Charadrius obscurus aquilonius) during the CV Rena oil-spill response. Wildlife Research **40**:624.

Gaudioso-Levita J, LaPointe D, Atkinson C, Egan A. 2015. Avian disease and mosquite vectors in the Kahuku Unit of Hawaii Volcanoes National Park and Ka’u Forest Reserve. Hilo, USA. Available from http://dspace.lib.hawaii.edu/handle/10790/2606 (accessed August 19, 2021).

Gaukler S, Linz G, Sherwood J, Dyer N, Bleier W, Wannemuehler Y, Nolan L, Logue C. 2009. Escherichia coli, Salmonella, and Mycobacterium avium subsp. paratuberculosis in wild European starlings at a Kansas cattle feedlot. Avian diseases **53**:544–551. Avian Dis.

German AC, Shankland GS, Edwards J, Flach EJ. 2002. Development of an indirect ELISA for the detection of serum antibodies to Aspergillus fumigatus in captive penguins. Veterinary Record **150**:513–518.

Gibson DJ, Nemeth NM, Beaufrère H, Varga C, Eagalle T, Susta L. 2019. Captive Psittacine birds in Ontario, Canada: a 19-Year retrospective study of the causes of morbidity and mortality. Journal of Comparative Pathology **171**:38–52.

Gibson TCM. 2010. The seasonality of parasites in Illinois house sparrows (Passer domesticus): Effect of stress on infection parameters. Eastern Illinois University, Charleston, IL, USA.

Globokar M, Fischer D, Pantchev N. 2017. Occurrence of endoparasites in captive birds between 2005 to 2011 as determined by faecal flotation and review of literature. Berliner und Münchener Tierärztliche Wochenschrift **130**:461–473.

Goetting V, Lee KA, Woods L, Clemons K V., Stevens DA, Tell LA. 2013. Inflammatory marker profiles in an avian experimental model of aspergillosis. Medical Mycology **51**:696–703.

Golemansky V. 2011. Coccidian parasites (Apicomplexa) of penguins (Pygoscelis ssp.) from Livingston Island and King George Island, the Antarctic. Polish Polar Research **32**:263–268.

González-Hein G, González C, Hidalgo H. 2008. Case report: an avian pox outbreak in captive psittacine birds in Chile. Journal of Exotic Pet Medicine **17**:210-215.

Graczyk TK, Cockrem JF. 1995. Aspergillus spp. seropositivity in New Zealand penguins. Mycopathologia **131**:179–184.

Hartup BK. 2008. Surveillance for West Nile Virus at the International Crane Foundation 2000-2004. Pages 111–114 in M. J. Folk and S. A. Nesbitt, editors. Proceedings of the Tenth North American Crane Workshop. North American Crane Working Group, Zacatecas City, Zacatecas, Mexico.

Hereba AM, Shathele MS, AHamouda M. 2016. Studies on experimental infection with Aspergillus fumigatus in ostrich chicks. The Journal of Animal & Plant Sciences **26**:1609–1613.

Herrera I, Khan MS, Kaleta E, Müller H, Dolz G, Neumann U. 2001. Serological status for *Chlamydophila psittaci*, Newcastle disease virus, avian polyoma virus, and Pacheco disease virus in scarlet macaws (*Ara macao*) kept in captivity in Costa Rica. Journal of Veterinary Medicine, Series B **48**:721-726.

Hodge P, Sandy J, Noormohammadi A. 2019. Avian mycobacteriosis in captive brolgas (Antigone rubicunda). Australian Veterinary Journal **97**:81–86.

Höfle U, Gortázar C, Ortiz J-A, Knispel B, Kaleta E. 2004. Outbreak of trichomoniasis in a woodpigeon (*Columba palumbus*) wintering roost. European Journal of Wildlife Research **50**:73-77.

Hofmeister EK, Jankowski MD, Goldberg D, Franson JC. 2016. Survey for West Nile Virus antibodies in wild ducks, 2004-06, USA. Journal of Wildlife Diseases **52**:354–363.

Hubálek Z, Halouzka J, Juřicová Z, Šikutová S, Rudolf I, Honza M, Janková J, Chytil J, Marec F, Sitko J. 2008a. Serologic survey of birds for West Nile flavivirus in southern Moravia (Czech Republic). Vector-Borne and Zoonotic Diseases **8**:659-666.

Hubálek Z, Wegner E, Halouzka J, Tryjanowski P, Jerzak L, Šikutová S, Rudolf I, Kruszewicz AG, Jaworski Z, Wlodarczyk R. 2008b. Serologic survey of potential vertebrate hosts for West Nile virus in Poland. Viral Immunology **21**:247-254.

Hukkanen RR, Richardson M, Wingfield JC, Treuting P, Brabb T. 2003. Avipox sp. in a colony of gray-crowned rosy finches (*Leucosticte tephrocotis*). Comparative medicine **53**:548-552.

Hull J, Ernest H, Hull J, Hull A, Reisen W, Fang Y, Ernest H. 2006. Variation of West Nile virus antibody prevalence in migrating and wintering hawks in central California. The Condor **108**:435-439.

Hull JM, Keane JJ, Tell L, Ernest HB. 2010. West Nile Virus antibody surveillance in three Sierra Nevada raptors of conservation concern. The Condor **112**:168–172.

Ibañez L, Gamboa MI, Fiorini VD, Montalti D. 2016. Coproparasitological study of European starling nestlings (Sturnus vulgaris) in Argentina. Turkish Journal of Zoology **40**:641–644.

Johnson B, Castro A. 1986. Canary pox causing high mortality in an aviary. Journal of the American Veterinary Medical Association **189**:1345-1347.

Kabeya H, Sato S, Maruyama S. 2015. Prevalence and characterization of Chlamydia DNA in zoo animals in Japan. Microbiology and immunology **59**:507-515.

Kane OJ, Uhart MM, Rago V, Pereda AJ, Smith JR, Van Buren A, Clark JA, Boersma PD. 2012. Avian pox in Magellanic penguins (*Spheniscus magellanicus*). Journal of wildlife diseases **48**:790-794.

Keeler SP, Yabsley MJ, Fox JM, McGraw SN, Hernandez SM. 2011. Isospora troglodytes n. sp. (Apicomplexa: Eimeriidae), a new coccidian species from wrens of Costa Rica. Parasitology Research 2011 110:5 **110**:1723–1725.

Keeler SP, Yabsley MJ, Gibbs SEJ, McGraw SN, Hernandez SM. 2012. A New Isospora Species of Passerines in the Family Turdidae from Costa Rica. Journal of Parasitology **98**:167–169.

Keeler SP, Yabsley MJ, Adams HC, Hernandez SM. 2014. A Novel Isospora Species (Apicomplexa: Eimeriidae) from Warblers (Passeriformes: Parulidae) of Costa Rica. Journal of Parasitology **100**:302–304.

Kilpatrick AM, Peters RJ, Dupuis II AP, Jones MJ, Daszak P, Marra PP, Kramer LD. 2013. Predicted and observed mortality from vector-borne disease in wildlife: West Nile virus and small songbirds. Biological conservation **165**:79-85.

Komar N, Langevin S, Hinten S, Nemeth N, Edwards E, Hettler D, Davis B, Bowen R, Bunning M. 2003. Experimental infection of North American birds with the New York 1999 strain of West Nile virus. Emerging infectious diseases **9**:311.

Komar N, Panella NA, Langevin SA, Brault AC, Amador M, Edwards E, Owen JC. 2005. Avian hosts for West Nile virus in St. Tammany Parish, Louisiana, 2002. American Journal of Tropical Medicine and Hygiene **73**:1031.

Komar N, Panella NA, Young GR, Brault AC, Levy CE. 2013. Avian hosts of West Nile Virus in Arizona. The American Journal of Tropical Medicine and Hygiene **89**:474. The American Society of Tropical Medicine and Hygiene.

Križek I, Horvatek D, Gottstein Ž, Steiner Z, Galović D, Ervaćinović Ž, Prukner-Radovčić E. 2012. Epidemiological study of Chlamydophila psittaci in pet birds in Croatia. Acta veterinaria **62**:325-331.

Krone O, Altenkamp R, Kenntner N. 2005. Prevalence of *Trichomonas gallinae* in northern goshawks from the Berlin area of northeastern Germany. Journal of Wildlife Diseases **41**:304-309.

Kulich P, Roubalová E, Dubská L, Sychra O, Šmíd B, Literák I. 2008. Avipoxvirus in blackcaps (*Sylvia atricapilla*). Avian Pathology **37**:101-107.

Kwan JL, Kluh S, Madon MB, Reisen WK. 2010. West Nile Virus emergence and persistence in Los Angeles, California, 2003–2008. The American Journal of Tropical Medicine and Hygiene **83**:400. The American Society of Tropical Medicine and Hygiene.

Kwan JL, Kluh S, Reisen WK. 2012. Antecedent avian immunity limits tangential transmission of West Nile Virus to humans. PLOS ONE **7**:e34127.

Lachish S, Lawson B, Cunningham AA, Sheldon BC. 2012. Epidemiology of the emergent disease Paridae pox in an intensively studied wild bird population. PLoS One **7**:e38316.

Lagerquist JE, Davison M, Foreyt WJ. 1994. Lead poisoning and other causes of mortality in Trumpeter (Cygnus buccinator) and Tundra (C. columbianus) swans in Western Washington. Journal of Wildlife Diseases **30**:60–64.

Le Loc'h G, Paul M, Camus‐Bouclainville C, Bertagnoli S. 2016. Outbreaks of Pox Disease Due to Canarypox‐Like and Fowlpox‐Like Viruses in Large‐Scale Houbara Bustard Captive‐Breeding Programmes, in Morocco and the United Arab Emirates. Transboundary and emerging diseases **63**:e187-e196.

Levine RS, Mead DG, Kitron UD. 2013. Limited spillover to humans from West Nile Virus viremic birds in Atlanta, Georgia. Vector Borne and Zoonotic Diseases **13**:812.

Loss SR, Hamer GL, Walker ED, Ruiz MO, Goldberg TL, Kitron UD, Brawn JD. 2009. Avian host community structure and prevalence of West Nile virus in Chicago, Illinois. Oecologia **159**:415–424.

Ludwig GV, Calle PP, Mangiafico JA, Raphael BL, Danner DK, Hile JA, Clippinger TL, Smith JF, Cook RA, McNamara T. 2002. An outbreak of West Nile virus in a New York City captive wildlife population. The American journal of tropical medicine and hygiene **67**:67-75.

Lugarini C, Albuquerque MCF de, Vanstreels RET, Roos AL, Silva JCR, Oliveira JB de. 2018. Endoparasites in birds of Guaribas Biological Reserve, Atlantic Forest, Paraíba State, Brazil. Ciência Animal Brasileira **19**.

MacDonald AM, Jardine CM, Rejman E, Barta JR, Bowman J, Cai HY, Susta L, Nemeth NM. 2019. High prevalence of Mycoplasma and Eimeria species in free-ranging Eastern wild turkeys (Meleagris gallopavo silvestris) in Ontario, Canada. Journal of Wildlife Diseases **55**:54–63.

Maluping RP, Oronan RB, Toledo SU. 2007. Detection of *Chlamydophila psittaci* antibodies from captive birds at the Ninoy Aquino Parks and Wildlife Nature Center, Quezon city, Philippines. Annals of Agricultural and Environmental Medicine **14**.

Marx M, Reiner G, Willems H, Rocha G, Hillerich K, Masello JF, Mayr SL, Moussa S, Dunn JC, Thomas RC. 2017. High prevalence of *Trichomonas gallinae* in wild columbids across western and southern Europe. Parasites & vectors **10**:1-11.

McKeon T, Dunsmore J, Raidal S. 1997. *Trichomonas gallinae* in budgerigars and columbid birds in Perth, Western Australia. Australian Veterinary Journal **75**:652-655.

McLean RG, Ubico SR, Docherty DE, Hansen WR, Sileo L, McNamara TS. 2001. West Nile virus transmission and ecology in birds. Annals of the New York Academy of Sciences **951**:54-57.

Melo AM, Silva Filho RP da, Poester VR, Fernandes CG, von Groll A, Stevens DA, Sabino R, Xavier MO. 2020. Aspergillosis in albatrosses. Medical Mycology **58**:852–855.

Michel F, Sieg M, Fischer D, Keller M, Eiden M, Reuschel M, Schmidt V, Schwehn R, Rinder M, Urbaniak S. 2019. Evidence for West Nile virus and Usutu virus infections in wild and resident birds in Germany, 2017 and 2018. Viruses **11**:674.

Millán J, Negre N, Castellanos E, de Juan L, Mateos A, Parpal L, Aranaz A. 2010. Avian mycobacteriosis in free-living raptors in Majorca Island, Spain. Avian pathology **39**:1–6.

Moens MA, Pérez‐Tris J, Milá B, Benítez L. 2017. The biological background of a recurrently emerging infectious disease: prevalence, diversity and host specificity of Avipoxvirus in wild Neotropical birds. Journal of Avian Biology **48**:1041-1046.

Morales-Betoulle M et al. 2013. West Nile virus ecology in a tropical ecosystem in Guatemala. The American journal of tropical medicine and hygiene **88**:116–126. Am J Trop Med Hyg.

Moravkova M, Lamka J, Kriz P, Pavlik I. 2011. The presence of Mycobacterium avium subsp. Avium in common pheasants (Phasianus colchicus) living in captivity and in other birds, vertebrates, non-vertebrates and the environment. Veterinarni Medicina **56**:333–343.

Nakeeb SM, Babus B, Clifton AY. 1981. Aspergillosis in the Peruvian Penguin (Spheniscus humboldti). The Journal of Zoo Animal Medicine **12**:54.

Naldo JL, Samour JH. 2004. Causes of Morbidity and Mortality in Falcons in Saudi Arabia. Journal of Avian Medicine and Surgery **18**:229–241.

Naugle DE, Aldridge CL, Walker BL, Doherty KE, Matchett MR, McIntosh J, Cornish TE, Boyce MS. 2005. West Nile virus and sage‐grouse: What more have we learned? Wildlife Society Bulletin **33**:616-623.

Nemeth N, Gould D, Bowen R, Komar N. 2006. Natural and experimental West Nile virus infection in five raptor species. Journal of Wildlife Diseases **42**:1-13.

Nemeth NM, Bosco-Lauth AM, Sciulli RH, Gose RB, Nagata MT, Bowen RA. 2010. Serosurveillance for Japanese Encephalitis and West Nile Viruses in Resident Birds in Hawai‘i. Journal of Wildlife Diseases **46**:659–664.

Nemeth NM, Gonzalez-Astudillo V, Oesterle PT, Howerth EW. 2016. A 5-Year Retrospective Review of Avian Diseases Diagnosed at the Department of Pathology, University of Georgia. Journal of Comparative Pathology **155**:105–120.

Nemeth NM, Williams LM, Bosco-Lauth AM, Oesterle PT, Helwig M, Bowen RA, Brown JD. 2021. West Nile Virus infection in ruffed grouse (Bonasa umbellus) in Pennsylvania, USA: A multi-year comparison of statewide serosurveys and vector indices. Journal of Wildlife Diseases **57**:51–59.

Niedringhaus KD et al. 2019. Trichomonosis due to Trichomonas gallinae infection in barn owls (Tyto alba) and barred owls (Strix varia) from the eastern United States. Veterinary Parasitology: Regional Studies and Reports **16**:100281.

Nugent G, Whitford E, Hunnam J, Wilson P, Cross M, Lisle G de. 2011. Mycobacterium avium subsp. paratuberculosis infection in wildlife on three deer farms with a history of Johne’s disease. New Zealand Veterinary Journal **59**:293–298.

O’Brien VA, Meteyer CU, Reisen WK, Ip HS, Brown CR. 2010. Prevalence and pathology of West Nile Virus in naturally infected house sparrows, western Nebraska, 2008. The American Journal of Tropical Medicine and Hygiene **82**:937. The American Society of Tropical Medicine and Hygiene.

Olias P, Gruber AD, Winfried B, Hafez HM, Lierz M. 2010. Fungal Pneumonia as a Major Cause of Mortality in White Stork (Ciconia ciconia) Chicks. Avian Diseases **54**:94–98.

Olsen G, Taylor J, Gee G. 1997. Whooping crane mortality at Patuxent Wildlife Research Center, 1982-95. Pages 243–248 in R. Urbanek and Stahlecker DW, editors. Proceedings of the Seventh North American Crane Workshop, 1996 Jan 10-13, Biloxi, Mississippi. North American Crane Working Group, Grand Island, NE. Available from https://digitalcommons.unl.edu/nacwgproc/230 (accessed August 31, 2021).

Ornelas-Eusebio E, Garcia-Espinosa G, Vorimore F, Aaziz R, Durand B, Laroucau K, Zanella G. 2020. Cross-sectional study on Chlamydiaceae prevalence and associated risk factors on commercial and backyard poultry farms in Mexico. Preventive Veterinary Medicine **176**:104922.

Oyarzún-Ruiz P, Cárdenas G, Fuente MCS la, Martin N, Mironov S, Cicchino A, Kinsella JM, Moreno L, González-Acuña D. 2021. Parasitic fauna of the invasive house sparrow (Passer domesticus) from Ñuble region, Chile: an example of co-introduced parasites. Revista Brasileira de Parasitologia Veterinária **30**:e004221.

Padilla LR, Santiago-Alarcon D, Merkel J, Miller RE, Parker PG. 2004. Survey for *Haemoproteus* spp., *Trichomonas gallinae*, *Chlamydophila psittaci*, and *Salmonella* spp. in Galapagos Islands columbiformes. Journal of Zoo and Wildlife Medicine **35**:60-64.

Palade E, Biró N, Dobos-Kovács M, Demeter Z, Mándoki M, Rusvai M. 2008. Poxvirus infection in Hungarian great tits (*Parus major*): case report. Acta Veterinaria Hungarica **56**:539-546.

Palmieri C, Roy P, Dhillon AS, Shivaprasad HL. 2013. Avian mycobacteriosis in Psittacines: A retrospective study of 123 cases. Journal of Comparative Pathology **148**:126–138.

Pérez Cordón G, Hitos Prados A, Romero D, Sánchez Moreno M, Pontes A, Osuna A, Rosales MJ. 2009. Intestinal and haematic parasitism in the birds of the Almuñecar (Granada, Spain) ornithological garden. Veterinary Parasitology **165**:361–366.

Piasecki T, Chrząstek K, Wieliczko A. 2012. Detection and identification of *Chlamydophila psittaci* in asymptomatic parrots in Poland. BMC Veterinary Research **8**:233.

Quillfeldt P, Schumm YR, Marek C, Mader V, Fischer D, Marx M. 2018. Prevalence and genotyping of *Trichomonas* infections in wild birds in central Germany. PloS one **13**:e0200798.

Redig PT, Fuller MR, Evans DL. 1980. Prevalence of Aspergillus fumigatus in free-living goshawks (Accipiter gentilis atricapillus). Journal of Wildlife Diseases **16**:169–174.

Reisen W, Fang Y, Martinez V. 2005. Avian host and mosquito (Diptera: Culicidae) vector competence determine the efficiency of West Nile and St. Louis encephalitis virus transmission. Journal of medical entomology **42**:367-375.

Reisen WK, Wheeler S, Armijos MV, Fang Y, Garcia S, Kelley K, Wright S. 2009. Role of communally nesting ardeid birds in the epidemiology of West Nile virus revisited. Vector-Borne and Zoonotic Diseases **9**:275-280.

Reisen WK, Padgett K, Fang Y, Woods L, Foss L, Anderson J, Kramer V. 2013. Chronic infections of West Nile Virus detected in California dead birds. Vector-Borne and Zoonotic Diseases **13**:401–405.

Rhim H, Cho Y-I, Jang H-J, Na K-J, Han J-I. 2018. High Prevalence of Mycobacterium avium subsp. paratuberculosis in Wild Ducks in the Middle Area of South Korea. Journal of Veterinary Clinics **35**:7–9.

Rodrigues MB et al. 2019. The vulnerable Sporophila frontalis (Verreaux) and Haplospiza unicolor Cabanis as new hosts for Isospora sporophilae Carvalho-Filho, Meireles, Ribeiro & Lopes, 2005 (Eimeriidae) in Brazil. Systematic Parasitology 2019 96:4 **96**:423–431.

Rosenfield RN, Bielefeldt J, Rosenfield LJ, Taft SJ, Murphy RK, Stewart AC. 2002. Prevalence of *Trichomonas gallinae* in nestling Cooper's Hawks among three North American populations. The Wilson Bulletin:145-147.

Ruiz-Martínez J, Ferraguti M, Figuerola J, Martínez-de la Puente J, Williams RAJ, Herrera-Duenas A, Aguirre JI, Soriguer R, Escudero C, Moens MAJ. 2016. Prevalence and genetic diversity of Avipoxvirus in house sparrows in Spain. PLoS One **11**:e0168690.

Russell RE, Franson JC. 2014. Causes of mortality in eagles submitted to the National Wildlife Health Center 1975–2013. Wildlife Society Bulletin **38**:697–704.

Samuel MD, Woodworth BL, Atkinson CT, Hart PJ, LaPointe DA. 2018. The epidemiology of avian pox and interaction with avian malaria in Hawaiian forest birds. Ecological Monographs **88**:621–637.

Sansano-Maestre J, Garijo-Toledo MM, Gómez-Muñoz MT. 2009. Prevalence and genotyping of *Trichomonas gallinae* in pigeons and birds of prey. Avian Pathology **38**:201-207.

Santos N, Jambas J, Monteiro A, Amaral J, Martins N, Garcia J, Fernández AM, Tyler KM, Almeida T, Abrantes J. 2019. *Trichomonas* Infection in a Community of Free-Ranging Domestic and Wild Columbiformes and Bonelli's Eagle (*Aquila fasciata*). Frontiers in veterinary science **6**:148.

Sattar A, Zakaria Z, Abu J, Aziz SA, Rojas-Ponce G. 2021. Isolation of Mycobacterium avium and other nontuberculous mycobacteria in chickens and captive birds in peninsular Malaysia. BMC Veterinary Research 2020 17:1 **17**:1–13.

Schmitz A, Korbel R, Thiel S, Wörle B, Gohl C, Rinder M. 2018. High prevalence of Mycobacterium genavense within flocks of pet birds. Veterinary Microbiology **218**:40–44.

Schoener ER, Alley MR, Howe L, Castro I. 2013. Coccidia species in endemic and native New Zealand passerines. Parasitology Research 2013 112:5 **112**:2027–2036.

Schulz JH, Bermudez AJ, Millspaugh JJ. 2005. Monitoring presence and annual variation of trichomoniasis in mourning doves. Avian Diseases **49**:387-389.

Shivaprasad H, Kim T, Tripathy D, Woolcock P, Uzal F. 2009. Unusual pathology of canary poxvirus infection associated with high mortality in young and adult breeder canaries (Serinus canaria). Avian pathology **38**:311-316.

Sidor IF, Pokras MA, Major AR, Poppenga RH, Taylor KM, Miconi RM. 2003. Mortality of common loons in New England, 1987 to 2000. Journal of Wildlife Diseases **39**:306–315.

Smits J, Tella JL, Carrete M, Serrano D, López G. 2005. An epizootic of avian pox in endemic short-toed larks (Calandrella rufescens) and Berthelot's pipits (Anthus berthelotti) in the Canary Islands, Spain. Veterinary Pathology **42**:59-65.

Souza MJ, Degernes LA. 2005. Mortality Due to Aspergillosis in Wild Swans in Northwest Washington State, 2000–02. Journal of Avian Medicine and Surgery **19**:98–106.

Stockdale JE, Dunn JC, Goodman SJ, Morris AJ, Sheehan DK, Grice PV, Hamer KC. 2015. The protozoan parasite *Trichomonas gallinae* causes adult and nestling mortality in a declining population of European Turtle Doves, *Streptopelia turtur*. Parasitology **142**:490-498.

Straub MH, Kelly TR, Rideout BA, Eng C, Wynne J, Braun J, Johnson CK. 2015. Seroepidemiologic survey of potential pathogens in obligate and facultative scavenging avian species in California. PLOS ONE **10**:e0143018.

Sukon P, Nam NH, Kittipreeya P, Sara-in A, Wawilai P, Inchuai R, Weerakhun S. 2021. Global prevalence of chlamydial infections in birds: A systematic review and meta-analysis. Preventive Veterinary Medicine **192**:105370.

Suksai P, Lorsunyaluck B, Dittawong P, Sanyathitiseree P, Lertwatcharasarakul P. 2016. Genetic detection and identification of *Chlamydophila psittaci* in captive Psittacine birds in Thailand. The Thai Journal of Veterinary Medicine **46**:67.

Tarello W. 2008. Prevalence and clinical signs of avipoxvirus infection in falcons from the Middle East. Veterinary dermatology **19**:101-104.

Tikasingh E, Worth C, Spence L, Aitken T. 1982. Avian pox in birds from Trinidad. Journal of wildlife diseases **18**:133-139.

Titilincu A, Mircean V, Bejan A, Iovu A, Ungureanu R, Cozma V. 2009. Prevalence of endoparasites in peacocks (Pavo cristatus). Revista Scientia Parasitologica **10**:101–105.

Tung KC, Liu JS, Cheng FP, Yang CH, Tu WC, Wang KS, Shyu CL, Lai CH, Chou CC, Lee WM. 2007. Study on the species-specificity of Isospora michaelbakeri by experimental infection. Acta Veterinaria Hungarica **55**:77–85.

van Riper III C, van Riper SG, Hansen WR. 2002. Epizootiology and effect of avian pox on Hawaiian forest birds. The Auk **119**:929-942.

Vianna Cardozo S, Pereira Berto B, Caetano I, Thomás A, Santos M, Pereira da Fonseca I, Gomes Lopes CW. 2019. Coccidian parasites from birds at rehabilitation centers in Portugal, with notes on Avispora bubonis in Old World. Brazilian Journal of Veterinary Parasitology **28**:187–193.

Villanúa D, Höfle U, Pérez-Rodríguez L, Gortázar C. 2006. *Trichomonas gallinae* in wintering common wood pigeons *Columba palumbus* in Spain. Ibis **148**:641-648.

Waeyenberghe L Van, Fischer D, Coenye T, Ducatelle R, Haesebrouck F, Pasmans F, Lierz M, Martel A. 2012. Susceptibility of adult pigeons and hybrid falcons to experimental aspergillosis. Avian Pathology **41**:563–567.

Walker BL, Naugle DE, Doherty KE, Cornish TE. 2007. West Nile virus and greater sage-grouse: estimating infection rate in a wild bird population. Avian Diseases **51**:691-696.

Wheeler SS, Barker CM, Fang Y, Veronica Armijos M, Carroll BD, Husted S, Johnson WO, Reisen WK. 2009. Differential impact of West Nile virus on California birds. The condor **111**:1-20.

Wheeler S, Woods L, Boyce W, Eckstrand C, Langevin S, Reisen W, Townsen A. 2014. West Nile Virus and non-West Nile Virus mortality and coinfection of American crows (Corvus brachyrhynchos) in California. Avian Diseases **58**:255–261.

Work TM, Dagenais J, Rameyer R, Breeden R. 2015. Mortality patterns in endangered Hawaiian geese (Nene; Branta sandvicensis). Journal of Wildlife Diseases **51**:688–695.

Williams RA, Truchado DA, Benitez L. 2021. A Review on the Prevalence of Poxvirus Disease in Free-Living and Captive Wild Birds. Microbiology Research **12**:403-418.

Wingate DB, Barker I, King N. 1980. Poxvirus infection of the white-tailed tropicbird (Phaethon lepturus) in Bermuda. Journal of Wildlife Diseases **16**:619-622.

Witte CL, Hungerford LL, Papendick R, Stalis IH, Rideout BA. 2008. Investigation of characteristics and factors associated with avian mycobacteriosis in zoo birds. Journal of Veterinary Diagnostic Investigation **20**:186–196.

Wrobel ER, Wilcoxen TE, Nuzzo JT, Seitz J. 2016. Seroprevalence of Avian Pox and Mycoplasma gallisepticum in Raptors in Central Illinois. Journal of Raptor Research **50**:289–294.

Xavier MO, Soares MP, Meinerz ARM, Nobre MO, Osório LG, da Silva Filho RP, Meireles MCA. 2007. Aspergillosis: A limiting factor during recovery of captive Magellanic penguins. Brazilian Journal of Microbiology **38**:480–484.

Yanga S, Martínez-Gómez J, Sehgal R, Escalante P. 2011. A preliminary survey for avian pathogens in Columbiform birds on Socorro Island, Mexico. Pacific Conservation Biology **17**:11–21

Yaremych SA, Warner RE, Mankin PC, Brawn JD, Raim A, Novak R. 2004. West Nile virus and high death rate in American crows. Emerging infectious diseases **10**:709.

Young LC, VanderWerf EA. 2008. Prevalence of avian pox virus and effect on the fledging success of Laysan Albatross. Journal of Field Ornithology **79**:93–98.

Zu Ermgassen EK, Durrant C, John S, Gardiner R, Alrefaei AF, Cunningham AA, Lawson B. 2016. Detection of the European epidemic strain of *Trichomonas gallinae* in finches, but not other non-columbiformes, in the absence of macroscopic disease. Parasitology **143**:1294-1300.

**Appendix S5:** Decision trees for remaining six hazards


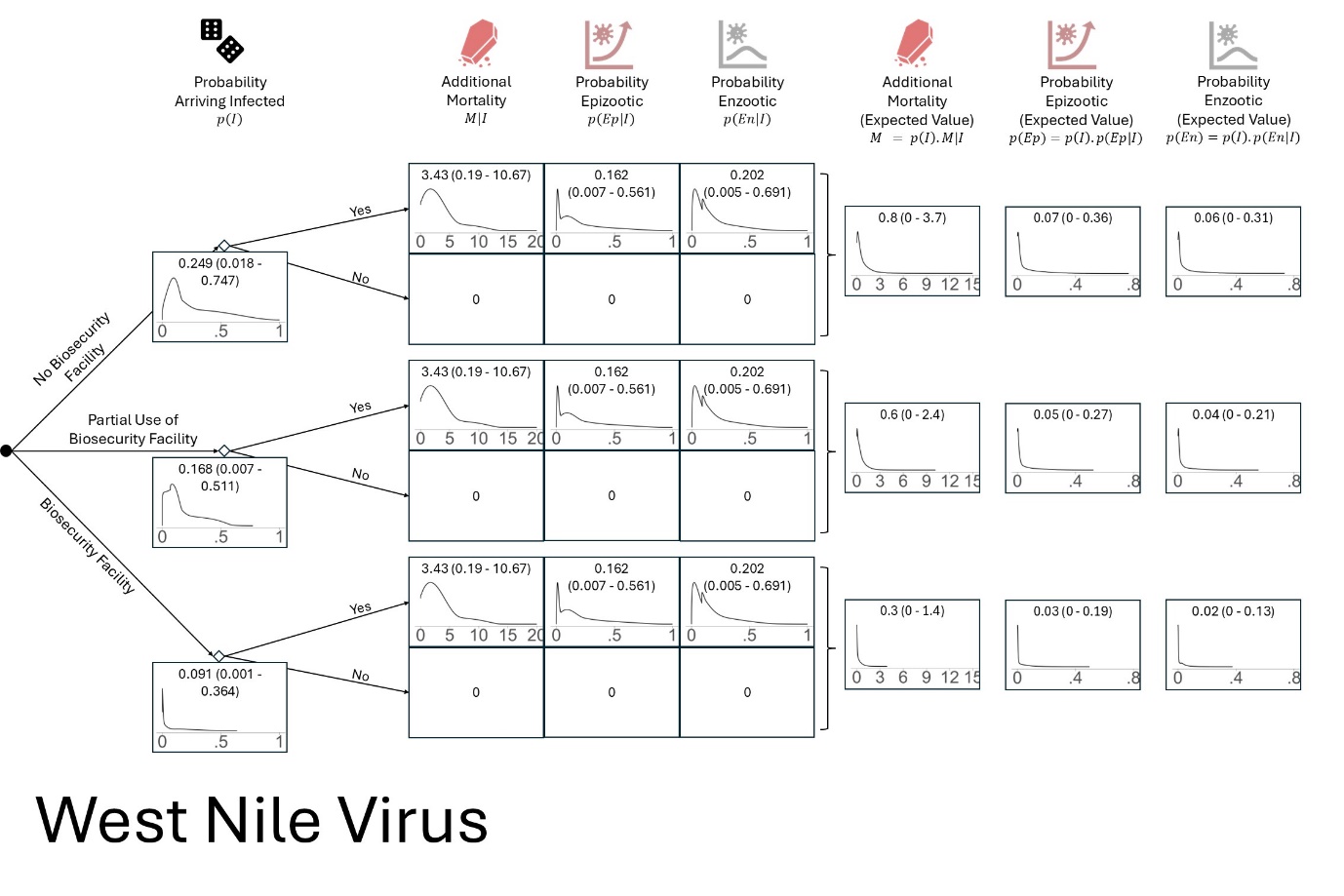

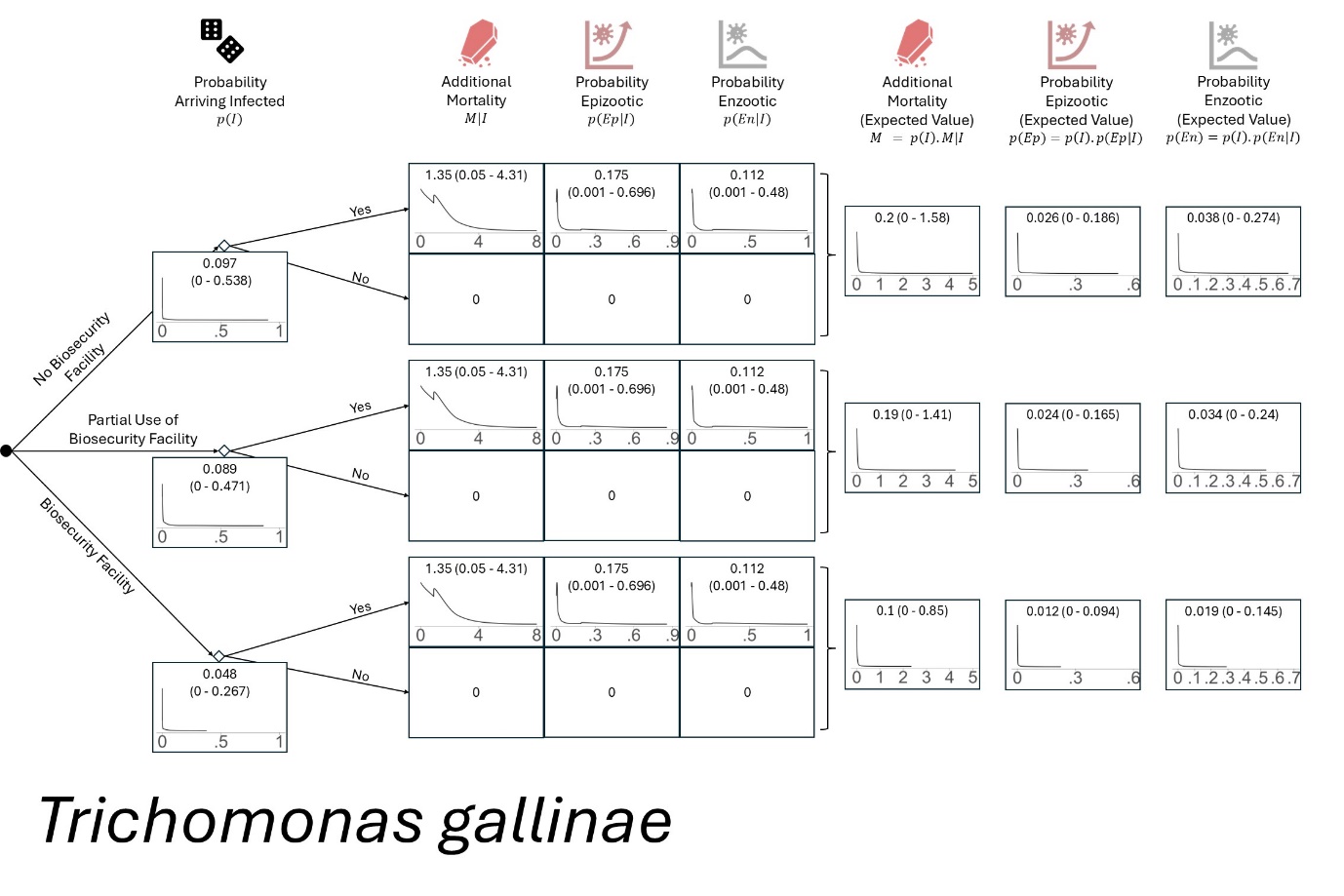

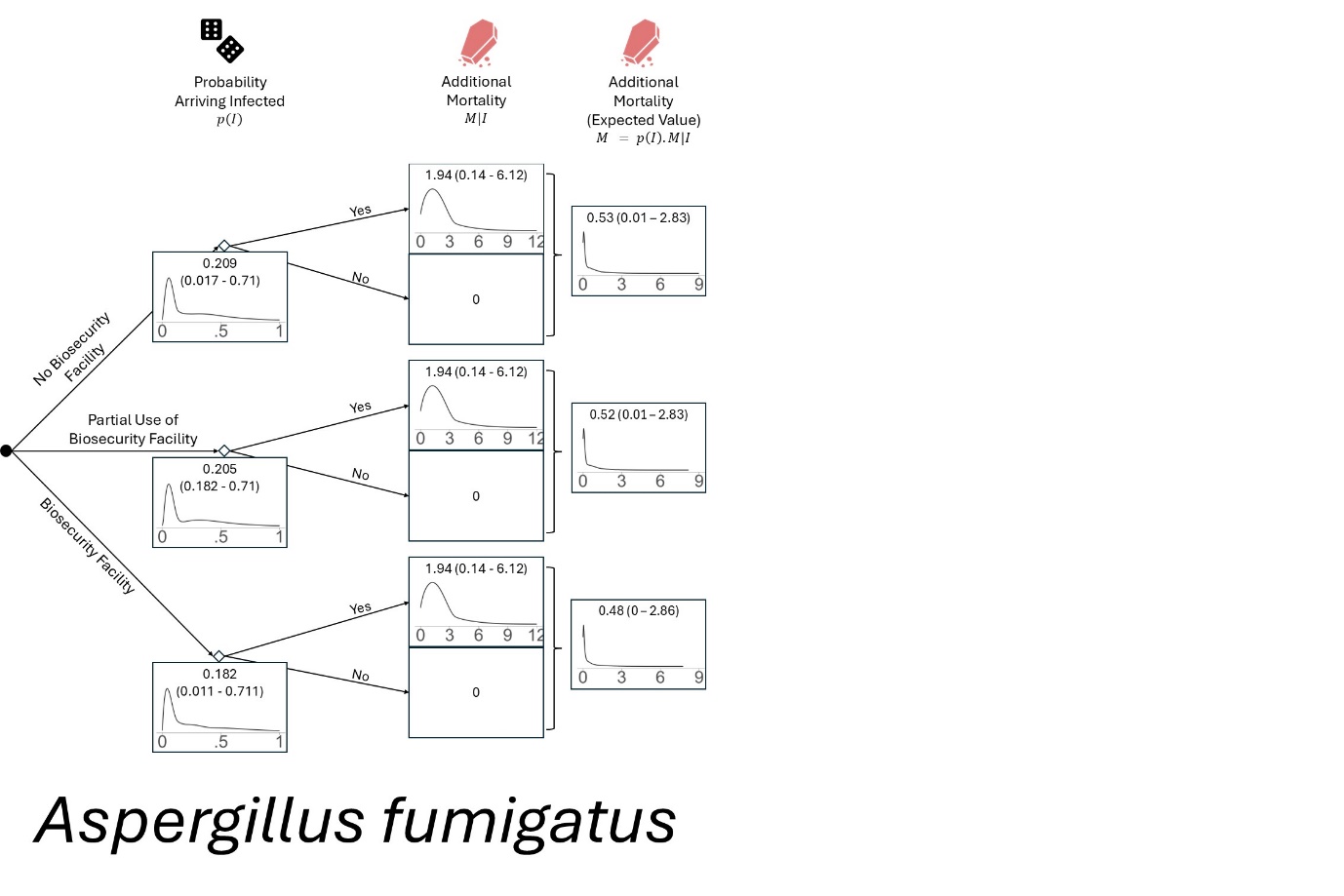

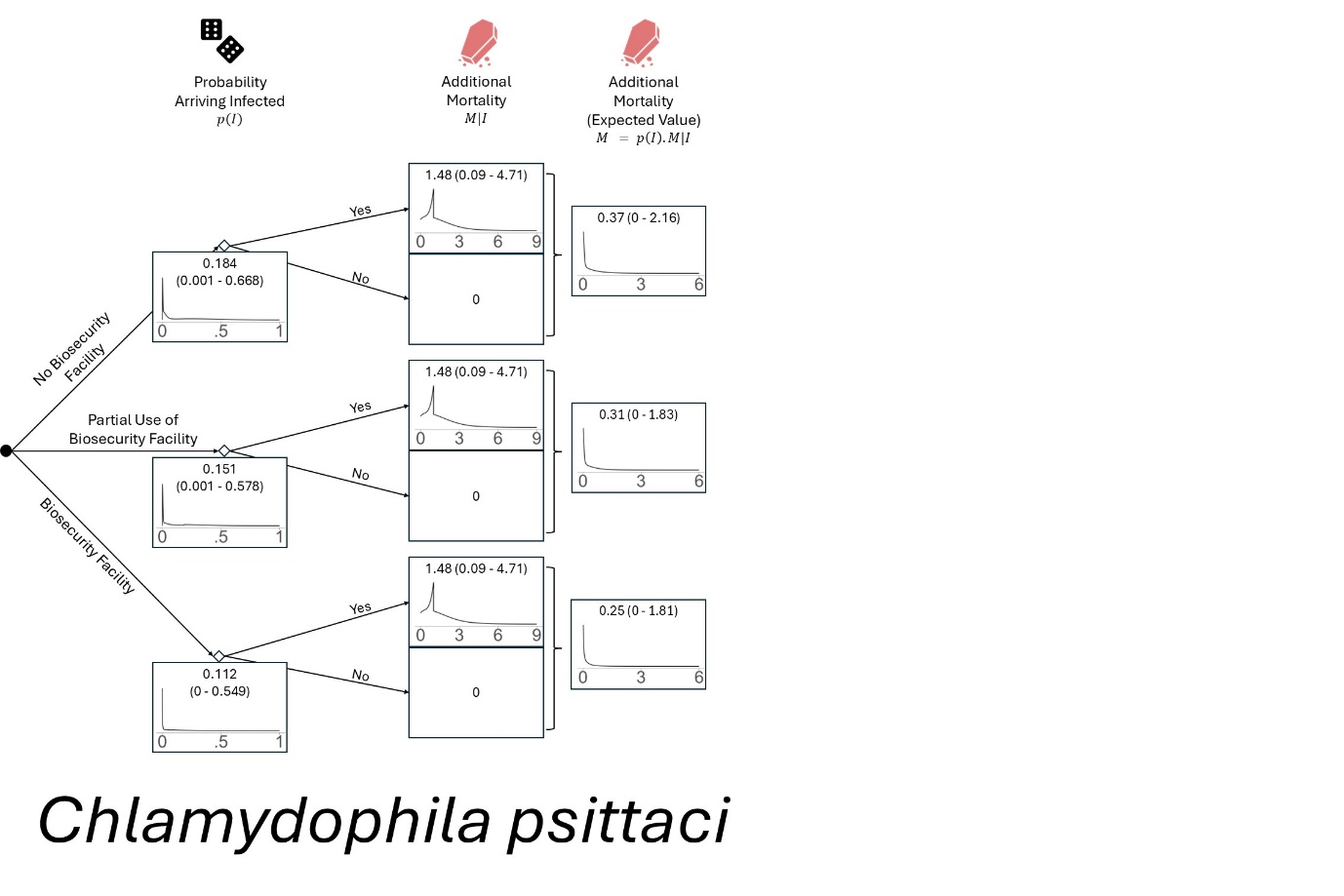

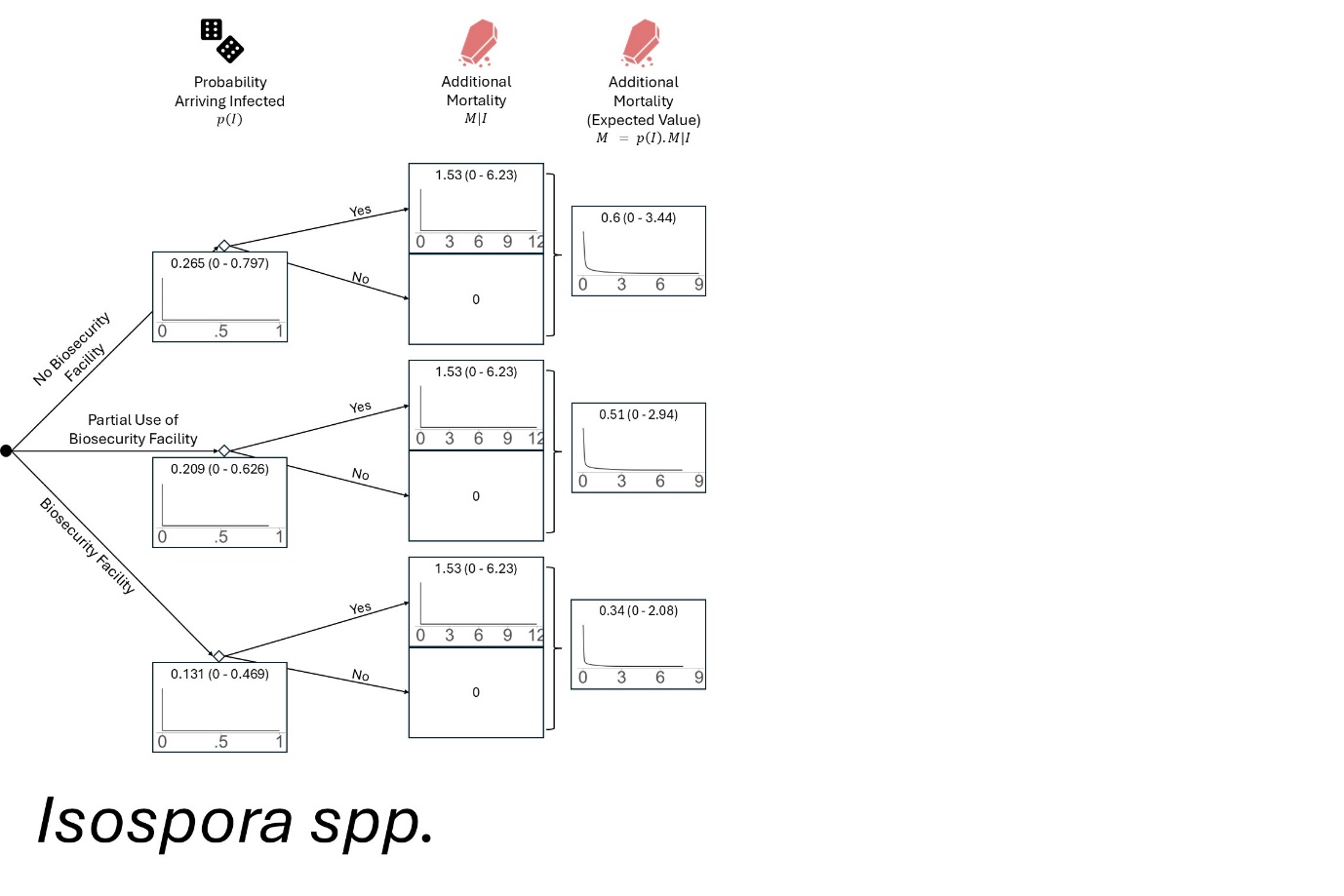

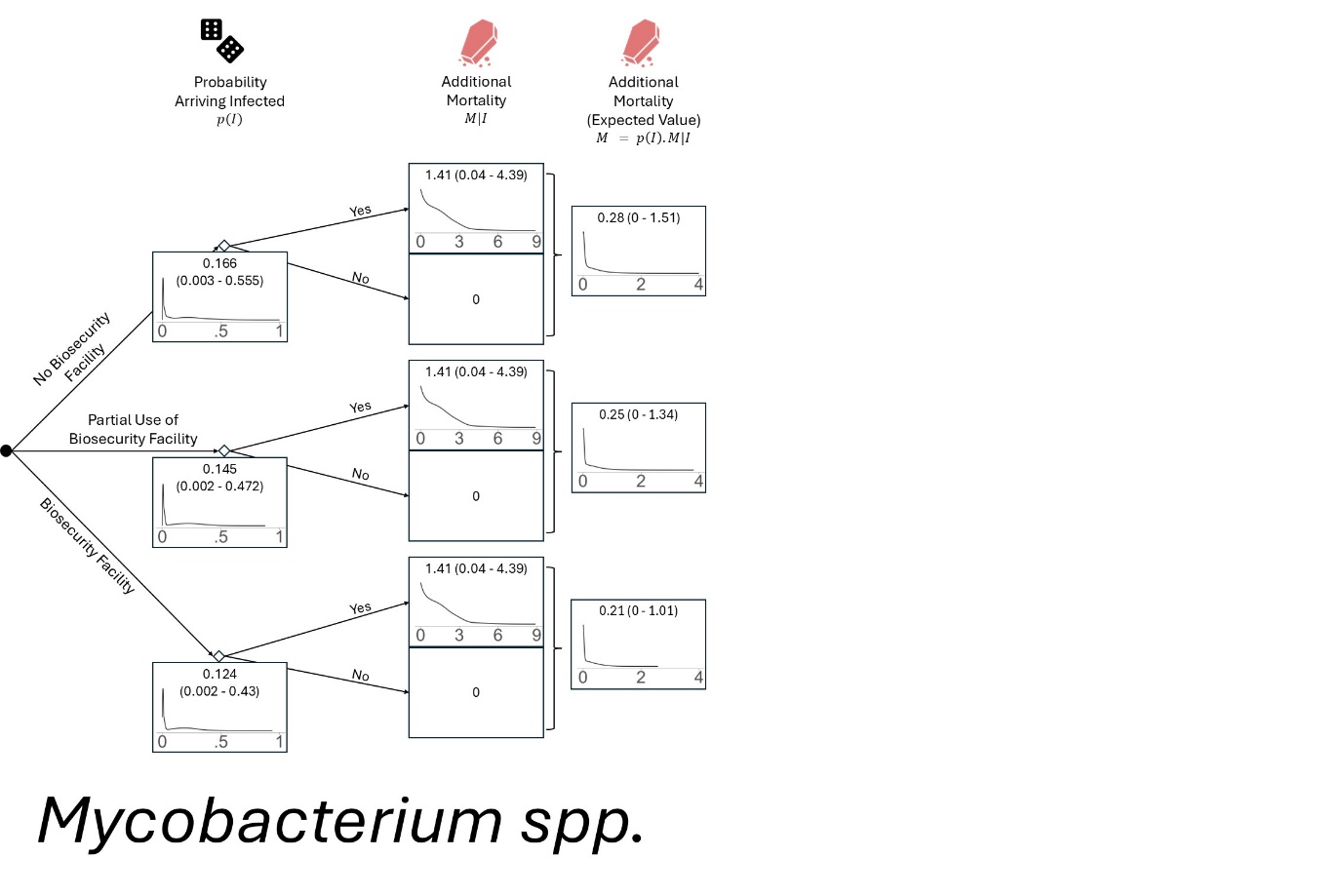

Supplement: Supplementary file 1 — Supporting Information [file COBI-40-e70292-s001.docx]
